# Development of Machine-Learning Models for Tinnitus-Related Distress Classification Using Wavelet-Transformed Auditory Evoked Potential Signals and Clinical Data

**DOI:** 10.3390/jcm12113843

**Published:** 2023-06-04

**Authors:** Ourania Manta, Michail Sarafidis, Winfried Schlee, Birgit Mazurek, George K. Matsopoulos, Dimitrios D. Koutsouris

**Affiliations:** 1 Biomedical Engineering Laboratory, School of Electrical and Computer Engineering, National Technical University of Athens, 15780 Athens, Greece; 2 Department of Psychiatry and Psychotherapy, University of Regensburg, 93053 Regensburg, Germany; 3 Institute for Information and Process Management, Eastern Switzerland University of Applied Sciences, 9001 St. Gallen, Switzerland; 4 Tinnitus Center, Charité—Universitätsmedizin Berlin, Freie Universität Berlin and Humboldt-Universität zu Berlin, 10117 Berlin, Germany

**Keywords:** tinnitus, auditory evoked potential, auditory brainstem response, auditory middle latency response, tinnitus-related distress, tinnitus handicap inventory, machine learning, classification modelling, wavelet scattering transform

## Abstract

Tinnitus is a highly prevalent condition, affecting more than 1 in 7 adults in the EU and causing negative effects on sufferers’ quality of life. In this study, we utilised data collected within the “UNITI” project, the largest EU tinnitus-related research programme. Initially, we extracted characteristics from both auditory brainstem response (ABR) and auditory middle latency response (AMLR) signals, which were derived from tinnitus patients. We then combined these features with the patients’ clinical data, and integrated them to build machine learning models for the classification of individuals and their ears according to their level of tinnitus-related distress. Several models were developed and tested on different datasets to determine the most relevant features and achieve high performances. Specifically, seven widely used classifiers were utilised on all generated datasets: random forest (RF), linear, radial, and polynomial support vector machines (SVM), naive bayes (NB), neural networks (NN), and linear discriminant analysis (LDA). Results showed that features extracted from the wavelet-scattering transformed AMLR signals were the most informative data. In combination with the 15 LASSO-selected clinical features, the SVM classifier achieved optimal performance with an AUC value, sensitivity, and specificity of 92.53%, 84.84%, and 83.04%, respectively, indicating high discrimination performance between the two groups.

## 1. Introduction

### 1.1. Tinnitus

Tinnitus is defined as the perception of a phantom sound and the patient’s reaction to it [1,2]. Most experts distinguish between subjective and objective tinnitus [2,3]. Tinnitus constitutes a common auditory symptom which can lead to severe impairment, especially when comorbidities are present [4]. In most sufferers, tinnitus is not due to medical causes, and there is no treatment available [5,6]. In many aspects, the presence of tinnitus is a heterogeneous and complex condition [7], and it can occur at any age with varying frequencies, intensities, and duration scales.

According to large, independent epidemiological studies, tinnitus affects more than 10% of the general population, whereas 1% of the population considers tinnitus to be their most serious health problem. The prevalence estimates in Europe are expected to double by 2050 [8,9,10]. Although significant scientific progress has been made in recent years [11,12,13,14,15], many patients with tinnitus remain either untreated or receive incomplete treatment. This situation contributes to increased complaints, prolonged suffering, social disengagement, excessive utilisation of healthcare services, and a complex network of referral pathways. Consequently, these result in substantial psychological and financial burdens at both national and global levels [1]. Despite its extremely high prevalence and socioeconomic burden, tinnitus remains a scientific and clinical mystery [16,17].

It is worth mentioning that the degree of tinnitus-related distress experienced by patients ranges from complete absence of discomfort to suicidal tendencies [18], resulting in a spectrum of different conditions that require distinct management and therapeutic approaches [19,20]. The various testing methods are unable to represent the degree to which tinnitus is troublesome on a case-by-case basis, although they are very useful in the diagnosis and planning of therapeutic interventions. For instance, patients with identical audiograms may have varying degrees of tinnitus perception in terms of intensity, severity, and induced disability [21,22]. This weakness in clinical and paraclinical examinations is well compensated by the use of tinnitus self-assessment questionnaires, which have been widely used in the clinical evaluation of tinnitus sufferers [7]. The use of these tests to objectively classify severity is considered to be an extremely useful tool in the hands of health professionals. Although the use of self-reported measures is considered a good practice, it is important to remember that self-assessment involves bias [1], which influences judgments and responses [19,23].

Several theories have been proposed to explain the mechanisms underlying tinnitus. Over the last decades, it has become apparent that tinnitus is closely related to hearing loss, but their degree of severity cannot be correlated. Moreover, the causal relevance of tinnitus is not limited to the cochlea, but probably involves many levels of the central auditory pathway, and even the central nervous system [24]. There is growing evidence that tinnitus etiopathogenetic mechanisms may be related to dysfunction or damage in parts of the auditory pathway [22,24]. This clinical hypothesis motivated auditory pathway testing through the use of auditory evoked potentials (AEPs) of tinnitus sufferers to assess and evaluate the severity of their tinnitus. In other words, given that the auditory pathway includes several stations involved in the conduction of sound, it is hypothesised that each of them could be associated with the occurrence of tinnitus; or, to state it simply, in order for an individual to hear tinnitus, one or more of the above stations or the connections between them must be affected.

### 1.2. Auditory Evoked Potentials (AEPs)

An AEP is an electrical signal produced by the brain in response to the presentation of a time-locked auditory stimulus [25,26]. The final AEP signal is composed of the average responses to thousands of stimulus repeats [27]. AEPs are a form of non-invasive and non-behavioural test whose main advantages are their simplicity, objectivity, reproducibility, and cost-effectiveness [22]. Based on a subject’s AEP response, audiologists are able to investigate potential obstacles along the neural pathways that lead to the brain. In addition, these signals may be useful for ruling out or confirming hearing impairments, particularly in neonates, and for medico-legal purposes to rule out benign tumours of the acoustic nerves, such as acoustic neuromas [28].

AEPs are classified as early (auditory brainstem responses—ABRs), middle (auditory middle latency responses—AMLRs), or late (auditory late latency responses—ALLRs) based on their occurrence time after the triggering stimulus [29].

**Figure 1:**
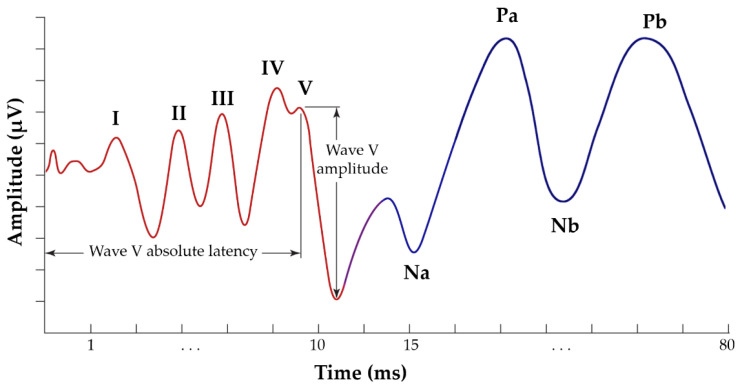
Typical annotated ABR signal, presenting the five waves of interest, from I to V (red waveform), and AMLR signal, presenting the four waves of interest: Na, Pa, Nb, and Pb (blue waveform).

In more detail, ABR is a sequence of acoustically stimulated signals that indicates synchronised neuronal activity along the neural pathways leading to the brain. It has a lengthy history of application and is regarded as one of the most reliable electrophysiological methods [30,31]. Within 10 milliseconds after the commencement of a moderately intense click stimulus, the derived ABR consists of five peaks coming from the auditory nerve and brainstem, annotated using Roman numerals (I through V) in capital letters (Figure 1). Wave I of the ABR reflects the activity of spiral ganglion cells in the distal eighth auditory nerve; wave II originates from the globular cells in the cochlear nucleus; wave III is generated by the cochlear nucleus’ spherical cells and globular cells; and waves IV and V are generated by the medial superior olive and its projections to the nuclei in the lateral lemniscus and the inferior colliculus [32,33]. Typically, these electrophysiological responses have an amplitude of less than one microvolt (μV) [28,34].

AMLR is typically recorded in a time window of 80 to 100 milliseconds and occurs around 12 to 60 milliseconds following the external stimulation. It is hypothesised that the thalamus and the auditory cortex are responsible for generating this response. AMLR is a waveform with four waves of interest: two troughs (Na and Nb) and two peaks (Pa and Pb) (Figure 1). The AMLR signal is sensitive to low frequencies, and there is typically a discrepancy of approximately 10 dB between the auditory thresholds measured behaviourally and electrophysiologically [35,36]. The shape of these waveforms varies considerably even among healthy individuals, with the Nb and Pb components appearing inconsistently [28].

ALLR is produced by non-primary cortical areas and is utilised to evaluate the integrity of the auditory system beyond the level of AMLR. It typically occurs 60 to 800 milliseconds after the external stimulus [37].

In brief, AEPs have predictable patterns and consist of discrete waves (peaks and troughs), which are the signal’s primary waves of interest and which are generated by specific stations along the auditory pathway [22]. The major metrics of an AEP are the latencies (the time between the initial auditory stimulus and the peak or trough of a waveform [31]), and absolute amplitudes related to the signal’s waves of interest [38]. Clinicians contemplate these measurements as metrics when interpreting these waveforms.

The objective identification and detection of bothersome tinnitus is a critical step in the proper management and administration of appropriate interventions or the combination of interventions for the patients. A detailed audiological evaluation, including ABR and AMLR analysis, could constitute an objective method for reflecting the functions of the cochlear or auditory nerve to auditory cortex. These electrophysiological methods are not currently included in routine clinical approaches, and have not been clearly correlated with the pathophysiology of tinnitus [39]. However, the utilisation of more objective data could have a strong influence on the way otolaryngologists investigate and understand tinnitus, focused towards an evidence-based approach.

### 1.3. The Scope of the Study

The major objective of this study is to assess the potential contribution of (early and middle) AEPs and clinical characteristics in determining the profile of patients with subjective tinnitus with reference to the severity or degree of distress manifested by their symptoms. For this purpose, we utilised the data collected in the context of a European tinnitus-related research project, named UNITI [18], and we developed a study workflow, which is briefly described below.

Initially, the patients’ AEP signals were used and their time-domain metrics were extracted. Consequently, statistically significant differences between the low- and high- tinnitus-distress sufferers were found. We then attempted to calculate metrics in the time-frequency domain using the wavelet scattering transform (WST) method and evaluate its performance, which was motivated by the former remarkable implementation of this method in electroencephalogram (EEG) applications [40]. The WST method is a mathematical technique used in signal processing and analysis. It is based on wavelets, which are functions capable of analysing signals at different scales and resolutions [40]. By applying this method, we were able to analyse the waveforms in a more comprehensive manner, capturing detailed information about their time-varying and frequency-related characteristics. To the best of our knowledge, although this method has proven to be very effective in solving EEG signal classification problems, it has not been used so far for AEP signals analysis. Subsequently, seeking to conduct more in-depth research and contribute to the profiling of patients with bothersome tinnitus, we integrated clinical characteristics of sufferers derived from their audiological examinations and responses to questionnaires. In each of the above stages, several known classifiers were implemented in various generated datasets in order to evaluate the effectiveness of the selected features in discriminating the level of discomfort caused by tinnitus and to determine the model with the highest classification performance. The classification algorithms applied were linear, radial, and polynomial support vector machines (SVM), random forests (RF), naive bayes (NB), neural networks (NN), and linear discriminant analysis (LDA). Overall, the various developed classification models included AEP metrics in time and time-frequency domains, as well as AEP metrics combined with clinical characteristics as features.

## 2. Materials and Methods

### 2.1. Data Origin, Recruitment Process, and Patient Characteristics

All data in this study were collected and selected from the tinnitus database of the European project “Unification of treatments and Interventions for Tinnitus patients” (Project Acronym: UNITI, Project Number: 848261, H2020-SC1-2019) [18]. The overall goal of UNITI is to deliver a predictive computational model based on existing and longitudinal data that attempts to determine which treatment approach is optimal for a particular patient, based on specific parameters [18]. The project combines clinical, epidemiological, medical, genetic, and audiological data, including electrophysiological signals (ABR and AMLR waveforms) gathered during a randomized clinical trial (RCT) (ClinicalTrials.gov Identifier: NCT04663828) [41]. Patient data in this study were derived from three different EU clinical centres (Hippocrateion General Hospital of Athens, Greece; Klinikum der Universitaet Regensburg, Germany; and Charité—Universitaetsmedizin Berlin, Germany).

The UNITI project consortium has confirmed the recruitment, inclusion, and exclusion criteria for the RCT participants (Table 1). All possible measures were taken to ensure that there was no discrimination in the recruitment, exclusion, or inclusion processes. Participants were not placed in any situation in which there was a possibility of physical, mental, or emotional harm or in any situation that threatened their physical or mental integrity. No monetary incentives were offered. 

Initially, a screening visit took place during which all the shortlisted participants were informed about the whole procedure and the recruited ones signed an informed consent form (ICF). The following procedure included a baseline visit and at least two follow-up visits (nine months after the baseline). During the baseline visit, a complete clinical assessment, collecting patients’ individual characteristics, history, and symptoms, was performed. Additionally, the participants responded to various health and tinnitus questionnaires, and electrophysiological measurements (ABR and AMLR) were conducted. In addition, any comorbidities or concomitant medications or treatments were recorded. All participating patients were required to make at least four visits to the respective clinical centres (1. screening, 2. baseline, 3. interim visit, and 4. final visit) over a period of at least 10 months [35]. Screening and baseline visits could be separated or combined. For the purposes of this study, data were derived exclusively from the screening and baseline visits so that the clinical data were time-corresponded to the waveform recordings.

### 2.2. Electrophysiological Measurements

All the waveforms were recorded and extracted with the Interacoustics Eclipse system (module EP25) [44]. This system offers the possibility of exporting raw measurements of auditory evoked potentials in .xml (Extended Markup Language) files. The exported files did not contain any patient data in violation of their privacy and security aspects, as stated by the EU General Data Protection Regulation (GDPR). The clinicians of the UNITI consortium pre-agreed on a standardised protocol for electrode placement and recording setups that was utilised by all clinical centres. This contributed to the uniformity of the collected data’s structure and quality, enabling comparisons and group analyses. 

For the recording of the ABR waveforms, the type of stimulus used was a click, and the repetition rate was 22 stimuli per second at an intensity level of 80 nHL. The recorded signal was filtered with a high-pass filter set at 33 Hz, 6 dB per octave, and a low-pass filter set at 1500 Hz, and the sample rate was 30 kHz. For recording the AMLR waveforms, the stimulus used was a 2 kHz tone burst with a duration of 28 sine waves, presented at a rate of 6.1 Hz per second and at an intensity level of 70 dB nHL. The recorded signal was filtered with a high-pass filter set at 10 Hz, 12 dB per octave, and a low-pass filter set at 1500 Hz, and the sample rate was 3 kHz. More details on the settings used for the stimulus and acquisition parameters of each test can be found in Table 2.

There were important parameters in the .xml files that had to be comprehended and used in order to reconstruct and visualise the ABR and AMLR records through the R programming language. Although there is not a publicly accessible *.xml* schema for the exported files, the manufacturer’s “Additional Information” manual for the EP25 module (https://www.manualslib.com/products/Interacoustics-Eclipse-Ep25-11647463.html, accessed on 5 December 2022) contains a description of the *.xml* header [45]. 

The R programming language and its accompanying packages, XML [46], xml2 [47], ggplot2 [48], signal [49], seewave [50], tuner [51], gsignal [52], MIMSunit [53], and base [54], were utilised to read the exported .xml files, rebuild the waveforms, and extract the associated data metrics.

### 2.3. Overall Study Workflow: From AEP Metrics to Classification Models Building

**Figure 2:**
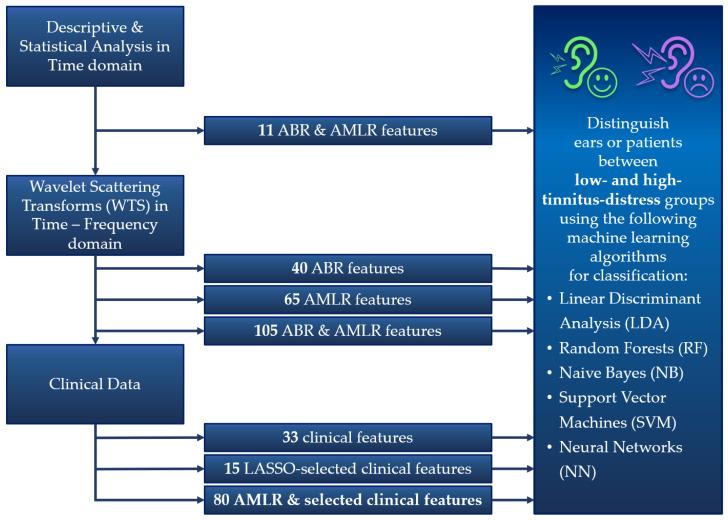
Overall Study Workflow.

The main purpose of this study was to utilize the AEP signals and various clinical characteristics in order to distinguish between low- and high-tinnitus-distress patient groups. Towards this goal, we extracted various metrics from the AEP signals and utilised them to build machine learning models. More specifically, we considered classical time-based features, which are also used as metrics by clinicians to evaluate these signals, and proceeded with more sophisticated wavelet scattering features in the time-frequency domain. In addition, clinical data from the UNITI project were integrated as input features into the classification models, aiming to highlight the predominant features for tinnitus-related distress. All the steps involved in our study’s workflow for building the various classification models are presented in Figure 2 and detailed in the following subsections.

### 2.4. Descriptive and Statistical Analyses in Time Domain

Statistical analyses were performed in continuation of previous studies [22,32,55] to determine the electrophysiological differences of patients suffering from subjective tinnitus, based on their manifested discomfort, ranging from mild/moderate distress to severe/catastrophic distress. We attempted to identify statistical differences on the time-domain core metrics of the AEP signals, i.e., the amplitudes and latencies of the waves of interest, which are also used by clinicians to interpret and evaluate the relevant waveforms. The metrics in which statistically significant differences were identified were selected as input features for the classification algorithms. A *p*-value of less than 0.05 was considered statistically significant, and parameter estimates were presented with their 95% confidence intervals (CI). Descriptive analyses included mean scores followed by standard deviations (SDs), as well as medians followed by minimum and maximum values for the continuous variables. T-tests for independent samples with equal or unequal variances (e.g., homo- and heteroscedastic samples) were used as appropriate to show whether the differences between the selected waveform metrics of compared groups were statistically significant.

Peaks and troughs of each waveform were annotated by the two automated tools for auditory evoked potential wave detection and annotation that were developed in the context of another study [56]. All statistical analyses and graphs were created using the R programming language, through the RStudio interface (version: 4.2.0) and its accompanying packages. Moreover, it was crucial to determine whether the samples followed a normal distribution. This can be examined using either analytic or graphical methods. However, the computed *p*-value is influenced by the sample size in analytical tests for normal distribution. In the present study, a large number of waveforms were employed. Hence, in order to avoid erroneous small values in the calculated *p*-values for testing normal distributions, graphical methods were selected as the most appropriate methods, particularly quantile–quantile plots (QQ plots). From the visualisation of the QQ plots, it emerged that the compared samples followed a normal distribution, which is why the parametric t-tests for independent samples (or unpaired t-tests) were chosen. Figure S1 shows indicatively the QQ plot of the dependent variable “Pb latency” for the obtained groups based on their THI scores. Through the leveneTest function of the car package (version: 3.1-0) [57,58], Levene’s tests were conducted to test the homogeneity of variance hypothesis for independent samples’ t-test. If Levene’s tests indicated unequal variances, then Welch’s t-tests (Welch’s unequal variances t-tests) were used to compare the groups, which was an alternative to the traditional analysis of parametric tests. For the two-tailed t-tests for independent samples, the function t.test of the stats package (version: 3.6.2) [59] was used, with a value of either FALSE or TRUE in the argument var.equal, depending on the Levene’s test results for variances. The t-test for independent groups determined whether there was a difference between two independent groups. However, the p-values do not indicate the strength of the difference, but only whether the difference is significant or not [60]. The effect size of the difference is widely used for meta-analysis or power analysis and demonstrates how “strong” the difference between the groups is. In this study, along with the statistical analyses, the effect sizes were calculated. To compute the standardized effect sizes with Cohen’s d, the cohen.d function of the effsize package (version: 0.8.1) [61] was used.

In the first phase, statistical analyses were performed using the THI questionnaire score [43] as the sole classification criterion. Therefore, all the electrophysiological data were divided into two groups, reflecting the patients’ degree of distress derived from tinnitus. As presented in Table 1, one criterion for the inclusion of participants in the study was whether they had a score of greater than or equal to 18 on the THI questionnaire. Therefore, even in the case of the minimum acceptable score, discomfort was present. A score of 48 was chosen as the optimal cut-off threshold between the two distress groups, after observing the participants’ score distribution and (a) seeking a numerical balance between the two groups being compared, and (b) isolating the patients with severe to catastrophic (bothersome) tinnitus in one category. Hence, the first group included all waveforms of participants with a THI score greater than or equal to 48, classifying them in the severe to catastrophic tinnitus-distressed group, while the second group included all waveforms of participants with a THI score less than 48, classifying them in the mild to moderate tinnitus-distressed group. The results of these statistical analyses were utilised in the feature selection phase for the time-domain waveform metrics. In particular, the waveform metrics for which statistically significant differences between the compared groups were identified (*p*-value < 0.05) were used as features in the relevant classification models. 

Subsequently, a more in-depth statistical analysis was performed. In this case, more stringent criteria were applied to categorise the compared groups, based on acoustic level and gender. These two factors influence waveform metrics, based on the literature [22,32,55]. In particular, based on the audiometric thresholds at octave frequencies ranging between 250 Hz and 8 kHz, each ear was classified into one of three groups: normal hearing [0–20 dB HL], mild hearing loss [21–60 dB HL], and severe hearing loss [>61 dB HL]. Each group was then divided into four sub-groups according to the participant’s gender and score on the THI questionnaire. Therefore, a total of twelve sub-groups emerged. Then, descriptive and statistical analyses of the groups under comparison were undertaken.

### 2.5. Wavelet Scattering Transform in Time–Frequency Domain

The use of wavelet scattering transform (WST) for signal processing was introduced by Prof. Stéphane Mallat and has grown in popularity over recent decades [62,63]. Mallat et al. proved the WST’s ability to retrieve trustworthy information at various scales. This transform may yield time and frequency resolutions that are translation-invariant, deformation-stable, and maintain high-frequency classification information [64,65]. In other literature, it has been demonstrated that wavelet scattering coefficients are more insightful than Fourier transform coefficients when handling signals with brief variation or minor deformation and rotation invariant [62,66,67]. In addition, WST combines the benefits of conventional and deep learning methodologies [68], integrating common properties of multiscale contractions, the linearization of hierarchical symmetries, and sparse representation.

In summary, wavelet scattering networks perform three main tasks that comprise a deep network: convolution, non-linearity, and pooling. In this case, convolution is performed by wavelets, the modulus operator serves as a non-linearity, and filtering with wavelet low-pass filters is analogous to pooling [69]. The filters in the scattering network are established a priori, rather than learnt, compared to deep convolutional networks, which is a fundamental contrast between the two frameworks. As the scattering transform is not required to learn the filters, it can be often used successfully in situations where there is a shortage of training data [69]. Scattering networks enable the automated generation of features that minimise differences within a class, while maintaining discriminability across classes [64,69]. Therefore, the WTS can be used as an automatic robust feature extractor for classification, since its extracted features are known to be translation or shift invariant, and stable against time-warping deformations. The features extracted using the scattering framework can then be inputted into a deep-learning or machine-learning model for classification. Consequently, we conducted AEP signal analysis through the use of wavelet scattering networks. To the best of our knowledge, this has not been carried out before in any related studies. 

In more depth, the WST is a cascaded decomposition and convolution of a signal with wavelets, followed by complex modulus and local averaging [70]. The convolution of the signal x with the dilated mother wavelet ψ with a centre frequency of λ (i.e., x*ψ_λ_) is the initial step in computing the WST. The convolved signal oscillates on a 2^j^ scale, and averaging such a signal yields zero. The convolved signal (i.e., |x*ψ_λ_|) is subjected to a nonlinear (modulus/rectifier) operator in order to eliminate these oscillations (i.e., complex phase). This operation increases the frequency of a signal by a factor of 2 and can be used to compensate for information losses due to downsampling. Finally, a time-average, low-pass filter φ is applied to the absolute convolved signal (i.e., |x* ψ_λ_|*φ). Therefore, during a half-overlapping time window of size 2^j^, the first-order scattering coefficients are defined as the average absolute amplitudes of wavelet coefficients for any scale (i.e., 1≤ j ≤ J) and are acquired with:

S_1x_ (t, λ1) = |x*ψ_λ1_|*φ. (1)

The repetition of the aforementioned procedures applied to each of |x*ψ_λ1_| results in the calculation of the second-order scattering coefficients, i.e.,

S_2x_ (t, λ1, λ2) = ||x*ψ_λ1_|*ψ_λ2_|*φ. (2)

By repeating the procedure above, the higher-order (i.e., m ≥ 2) wavelet scattering coefficients can be estimated as

S_mx_ (t, λ1, …, λm) = |||x*ψ_λ1_|*ψ_λ2_|…*ψ_λm_|*φ. (3)

The zero-order scattering coefficients, which are derived with a time-average S_0x_ (t) = x*φ, explain the local translation invariance of the signal. The averaging operation at each stage eliminates the high-frequency contents of the convolved signal, which can be recovered by convolving the signal with the wavelet in the subsequent stage. With each additional layer, the energy of the scattering coefficients diminishes, with the top two levels holding 99% of the energy [71]. Additionally, Ahmad et al. [72] determined that an order of 2 is ideal for using WST to extract features from EEG.

**Figure 3:**
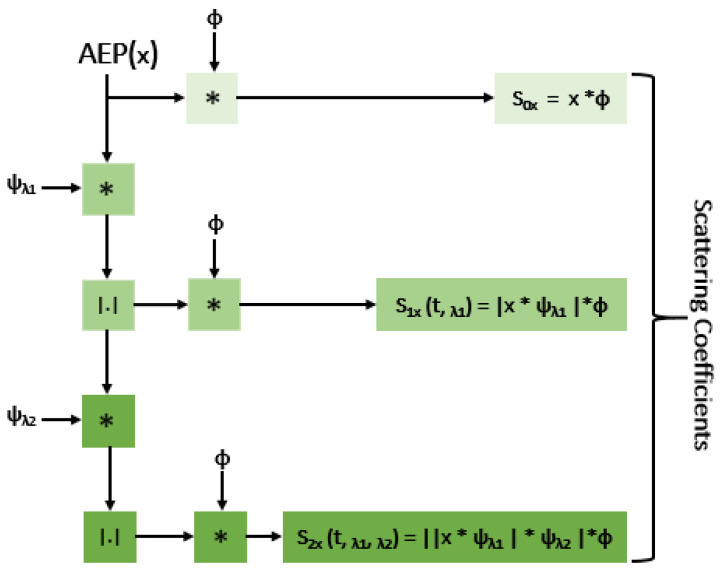
Schematic illustrating the feature extraction from AEP signals using second-order WST. S_0x_, S_1x_, and S_2x_, denote the 0th (time-averaged or low-pass filtered), 1st, and 2nd order scattering coefficients of WST, respectively. * and |.| represent the convolution and modulus operators, respectively.

The process for calculating the WST coefficients at each level is shown in Figure 3, and the aggregated coefficients were then employed as the features.

In our study, one wavelet scattering network per each AEP subtype was used as a feature extractor for the classification purposes. These networks were applied in MATLAB and performed separately on each AEP subtype. A two-order wavelet scattering network was established in both cases. The steps carried out in order to extract the features from the two wavelet scattering networks are described hereafter.

Initially, the raw ABR and AMLR signals were extracted in order to be further analysed. There were 496 waveforms per test, and 450 samples, i.e., generated amplitude values (in µV), per waveform. Then, the signals were imported to MATLAB and the two wavelet-time scattering networks were constructed. Specifically, the key parameters for the scattering networks that needed to be specified were the scale of the time invariant [73] and the quality factor for the scattering filter band; that is, the number of wavelets per octave in each of the wavelet filter banks [68,74]. The wavelet transform discretises the scale using the specified number of wavelet filters [75]. In many applications, the cascade of two filter banks is sufficient to achieve good performance, and therefore we used this number in our two wavelet scattering networks.

After constructing the two scattering networks, we obtained the scattering coefficients for the two signal types. In both applications, the natural logarithms of the scattering coefficients were calculated [73,76]. Each WST yielded a scattering paths-by-scattering windows-by-signals tensor, which was transformed into a matrix that could be further fed into machine-learning classification algorithms. 

The wavelet-scattering method creates a large number of features, so we decided to limit the number of features for efficiency purposes. Wavelet scale averaging [77] was used in our analysis in order to minimise dimensionality by transforming the two-dimensional feature matrix of each signal into a one-dimensional feature vector to meet the input requirements of each classifier. In particular, this method averages over the wavelet scale (time window) dimensions and outputs the averaged coefficients.

For every AEP subtype, a unique dataset that included the final resulting wavelet scattering coefficients was extracted. The first dataset included the coefficients obtained from the WST of the ABR signals, and the second the coefficients obtained from the WST of the AMLR signals. Moreover, a combined dataset including the coefficients of both AEPs was created. The coefficients included in these datasets were used as features for the development of machine learning models (the 40, 65, and 105 ABR and AMLR features that are referenced in Figure 2).

### 2.6. Patients’ Clinical Data Integration

The clinical data were extracted from the UNITI database and were collected during the patients’ visits to the clinical centres. The dataset included 1302 patient visits, and every visit contained 630 variables, representing all the characteristics that could be filled in. The vast majority of these variables concerned answers to questionnaires, and the remaining columns concerned the results of the audiological measures (otological examination, pure tone audiometry [78], tinnitus pitch/loudness match, tinnitus maskability, residual inhibition). The clinical data contained information from a total of 27 questionnaires. The selection of questionnaires in each clinical centre varied according to the centre’s routine clinical practice and individual patient characteristics. In the context of the UNITI project, a consensus was reached on a common set of eight questionnaires used in daily clinical practice. These questionnaires included the Tinnitus Handicap Inventory (THI) [43], the Tinnitus Functional Index (TFI) [79], the Tinnitus Severity (TS) [80], the Mini-Tinnitus Questionnaire (Mini-TQ) [81,82], the Questionnaire on Hypersensitivity to Sound (GUF) [83,84], the Patient Health Questionnaire (PHQ-9) [85], the WHO Quality of Life Questionnaire (WHOQOL-BREF) [86], and the European School of Interdisciplinary Tinnitus Research Screening Questionnaire (ESIT-SQ) [87].

From the above-mentioned questionnaires, we selected a subset of them to be used as features in our classification models. Seeking to develop models which would be unaffected, as much as possible, by the subjectivity of the sufferers [88,89], we considered that questionnaires that resulted in a total unique score should be removed. Only the THI, which was used as the study’s dependent variable, and the GUF [90], which was not directly related to tinnitus and its severity assessment, were kept. Concerning the ESIT-SQ questionnaire, which is meant to collect medical characteristics (related and unrelated to tinnitus), only the questions completed by all three clinical centres were retained.

Hereinafter, the selection of the clinical data for building the classification models is described. The interim and final visits were excluded, selecting baseline values over screening visit values when both were available. The clinical variables with excessive missing values or common values that offered no meaningful information were also removed. In the remaining cases with missing data, the missing values were replaced with the means or most frequent values of the specific variables. Patients with no registered waveforms were also removed from the analysis.

It is noteworthy that some additional steps had to be performed on the data related to audiological tests. The values of the related variables had been derived separately for each ear, and they had to be modified in order to be utilised for the classification models performed on patients. For bilateral tinnitus patients, the values for “hearing loss”, “maximum tinnitus frequency”, “tinnitus matching loudness (in dB)”, and “minimal masking level (in dB)” were averaged between the two ears. For unilateral tinnitus patients, the above variables were assigned values based on the affected ear. Lastly, for head-derived tinnitus patients, the values from the ear(s) where tinnitus matching was detected were utilised. 

Following this procedure, a data frame including all the selected clinical features was created (Table S1). Every row in the data frame referred to a unique patient, and each column to a different clinical feature.

### 2.7. Building Classification Models

#### 2.7.1. Classification Models and Performance Evaluation

Based on the previous analysis conducted, we used the obtained AEP waveforms metrics as features for machine learning models, both in the time and time–frequency domains. The aim of the classifiers was to distinguish the tinnitus ears between low- and high-tinnitus-related distress groups. Various classifiers, including LDA [91], RF [92], NB [93], polynomial, linear, and radial SVM [94], and NN [95], were developed. The target variable was the THI score category (low or high THI score/tinnitus distress). The R programming language facilitated the training, testing, and validation of these classifiers using the caret package (version 6.0–94). Ten-fold cross-validation was implemented for all the models, reporting mean sensitivity, specificity [96], and AUC_ROC_ (Area under the ROC Curve) [97] for performance evaluation.

#### 2.7.2. Feature Selection in Clinical Data Using LASSO

To develop a classification model that was both precise and reliable, we investigated the most relevant patient data in terms of discriminating patients into low- and high-distress sufferers. Therefore, we utilised a feature selection method in order to define the most important clinical features and build a classification model based solely on patients’ clinical data. Subsequently, the least absolute shrinkage and selection operator (LASSO) method, with a 10-fold cross-validation and 100 iterations, was performed to select the most relevant features for the classification models. LASSO regression, implemented using the R package glmnet (version 4.1–4) [98], applies a penalty term to the regression coefficients, encouraging sparse solutions and effectively performing feature selection.

#### 2.7.3. Integration of AEP Metrics and Clinical Characteristics

In order to build a more accurate and robust classification model, we integrated the patients’ clinical data with the AEPs characteristics. The analysis considered each patient’s ear as an instance for our model, combining features from both clinical data and waveform metrics. Audiological measurements unrelated to tinnitus-matching ears were removed from the final dataset. 

## 3. Results

### 3.1. Descriptive and Statistical Analyses in the Time Domain

All waveforms used in the statistical analyses were obtained from 248 participants, of whom 101 were women, aged 24 to 76 years, with a mean age ± SD = 52.21 ± 12.16, and 147 were men, aged 25 to 77 years, with a mean age ± SD = 53.52 ± 12.76. For both AEP subtypes, two waveforms were recorded for each participant, one for each ear, resulting in a total of 496 waveforms per AEP subtype.

The subsequent two subsections expound upon the principal findings derived from the descriptive and statistical analyses of this study. Supplementary Materials, encompassing Tables S2–S9 and Figures S2–S5, provide further detailed information pertaining to the study’s results.

#### 3.1.1. Grouping Using the THI Score

**Figure 4:**
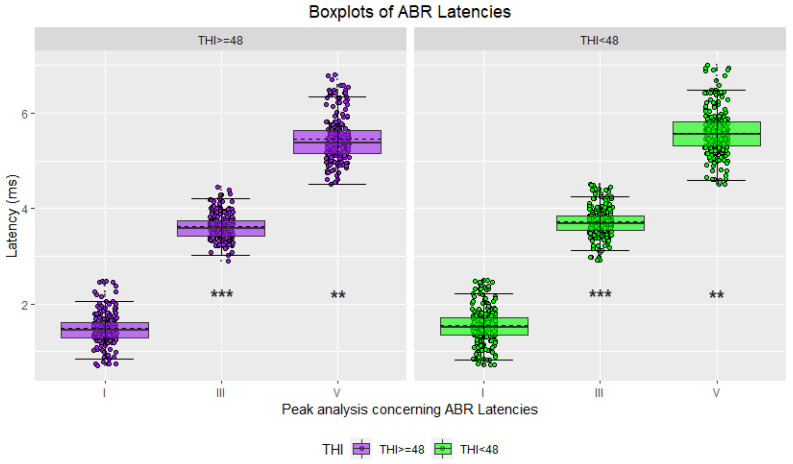
Boxplots of ABR waveform latencies based on tinnitus distress (in purple: severe/high tinnitus distress; in green: mild/moderate tinnitus distress; asterisks indicate significance: ** *p*-value ≤ 0.01; *** *p*-value ≤ 0.001).

**Figure 5:**
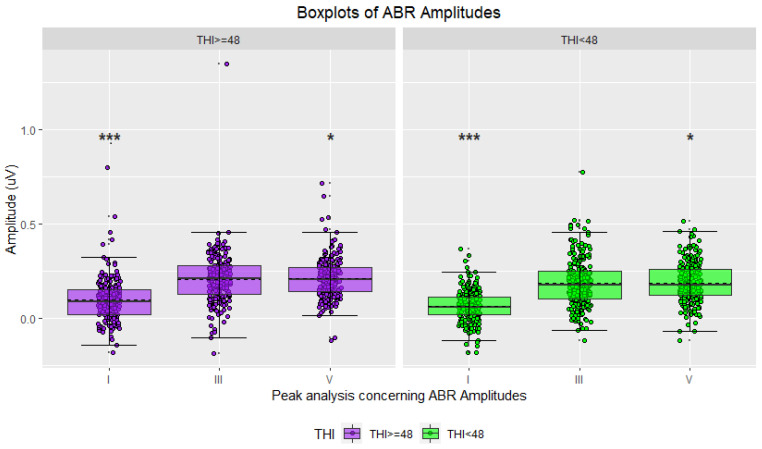
Boxplots of ABR waveform amplitudes based on tinnitus distress (in purple: severe/high tinnitus distress; in green: mild/moderate tinnitus distress; asterisks indicate significance: * *p*-value ≤ 0.05; *** *p*-value ≤ 0.001).

**Figure 6:**
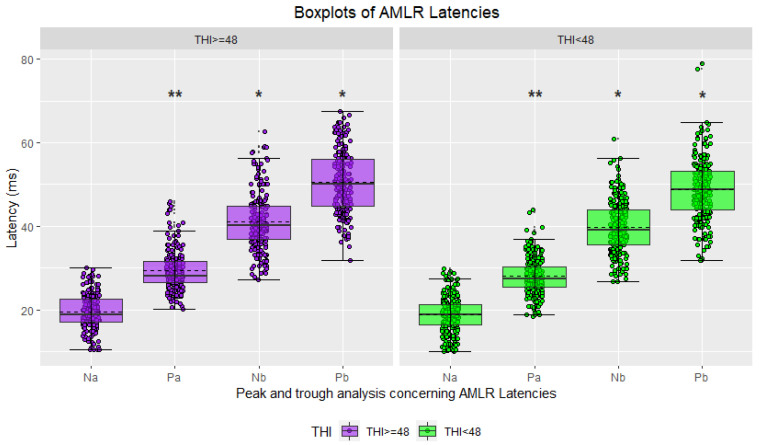
Boxplots of AMLR waveform latencies based on tinnitus distress (in purple: severe/high tinnitus distress; in green: mild/moderate tinnitus distress; asterisks indicate significance: * *p*-value ≤ 0.05; ** *p*-value ≤ 0.01).

**Figure 7:**
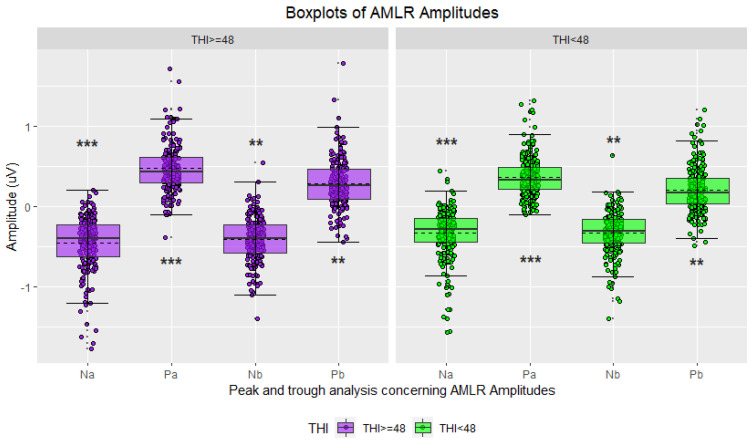
Boxplots of AMLR waveform amplitudes based on tinnitus distress (in purple: severe/high tinnitus distress; in green: mild/moderate tinnitus distress; asterisks indicate significance: ** *p*-value ≤ 0.01; *** *p*-value ≤ 0.001).

In the initial phase, statistical analyses were conducted using the THI score [43] as the only criterion to divide the study participants into two groups. Waveforms were classified accordingly, with 45.97% attributed to highly distressed tinnitus sufferers and the remaining 54.03% to moderately distressed tinnitus sufferers. Hereinafter, the results are shown as boxplots for the two groups’ amplitudes and latencies (Figure 4, Figure 5, Figure 6 and Figure 7), accompanied by tables including the descriptive statistics and the numerical results of the statistical analyses for all the pairs of compared groups (Tables S2–S5).

Descriptive statistics for the ABR waveforms indicated that sufferers with the lowest THI score had higher mean latencies for all three waves of interest (peak I, peak III, and peak V), while those with the highest THI score exhibited higher mean amplitudes (Figure 4 and Figure 5, Table S2). Conversely, the descriptive statistics for the AMLR waveforms revealed that sufferers in the highest THI score group displayed higher mean latencies and absolute mean amplitudes for all four waves of interest (Na trough, Pa peak, Nb trough, and Pb peak) (Figure 6 and Figure 7, Table S3).

The two-tailed t-tests for independent samples revealed that there were some statistically significant differences between the compared groups. Regarding ABR waveforms, significant differences were observed in peak III and peak V latencies, as well as in peak I amplitude (Figure 4 and Figure 5, Table S4). For AMLR waveforms, all metrics, except Na trough latency, showed statistically significant differences (Figure 6 and Figure 7, Table S5). In all cases, effect sizes indicated a small to medium effect.

#### 3.1.2. Grouping Using the THI Score Combined with Gender and Hearing Level

In a second stage, the compared groups consisted of waveforms from participants with matching hearing levels and genders, as these factors strongly influence waveform metrics. These factors, along with the THI score, were used as classification criteria for the patients’ waveforms, resulting in the emergence of twelve subgroups. 

Descriptive statistics for ABR waveforms indicated that subgroups with high THI scores (≥48) generally exhibited lower latency metrics compared to their corresponding subgroups with lower THI scores (<48). However, there were exceptions, including peak III latency in the “females with normal hearing” subgroup and peak V latency in the “males with severe hearing loss” subgroup. Conversely, amplitude metrics showed the opposite pattern, with subgroups with high THI scores showing higher amplitudes compared to their corresponding subgroups with low THI scores (Figures S2 and S3, Table S6). Exceptions were observed for peak I amplitude in the “males with severe hearing loss” subgroup and peaks III and V amplitudes in the “females with normal hearing” subgroup.

Descriptive statistics for AMLR waveforms revealed that subgroups with high THI scores generally exhibited equal or greater latency and amplitude metrics (absolute values) compared to their corresponding subgroups with low THI scores (Figures S4 and S5, Table S7). Exceptions were observed for Na and Pb latencies in the “females with severe hearing loss” subgroup, Na and Nb amplitudes in the “females with normal hearing” subgroup, and Nb amplitude in the “males with normal hearing” subgroup.

The two-tailed t-tests for independent samples revealed statistically significant differences between the compared groups. In the statistically significant differences identified, the effect sizes indicated a medium-to-large or large effect. For ABR waveforms, these differences concerned the following subgroups (Figures S2 and S3, Table S8):

- “Males with normal hearing” with respect to peak III latency and peak V amplitude;
- “Females with mild hearing loss” with respect to peak III and peak V latencies, and peak I amplitude;
- “Males with mild hearing loss” with respect to peak I amplitude;
- “Females with severe hearing loss” with respect to peak III and peak V latencies, and peak III amplitude;
- “Males with severe hearing loss” with respect to peak III latency.

Similarly, for AMLR waveforms, these significant differences concerned the following subgroups (Figures S4 and S5, Table S9):

- “Females with normal hearing” with respect to Nb trough and Pb peak latencies;
- “Females with mild hearing loss” with respect to Pa peak and Nb trough amplitudes;
- “Males with mild hearing loss” with respect to Nb trough latency and Na trough, Pa peak, and Nb trough amplitudes;
- “Males with severe hearing loss” with respect to Na trough amplitudes.

Tables S6–S9 provide descriptive statistics and the results of the statistical analyses for all compared groups. Correspondingly, Figures S2–S5 present boxplots illustrating the amplitudes and latencies of the compared metrics.

### 3.2. Wavelet Scattering Transform (WST) in the Time–Frequency Domain

#### 3.2.1. The Wavelet Scattering Transform Method

**Figure 8:**
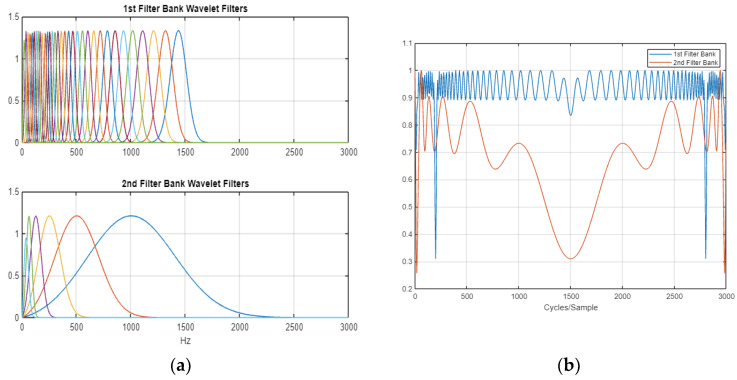
(**a**) Plot of the wavelet filters used in the first and second filter banks; (**b**) plot of the Littlewood–Paley sums of the filter banks.

In the context of this study, two filter banks were used in both wavelet scattering networks. In particular, we constructed two wavelet time scattering networks with default filter banks, i.e., eight wavelets per octave in the first filter bank (Q1) and one wavelet per octave in the second filter bank (Q2), in both networks [73]. Figure 8 depicts the quality factors (Q1 and Q2) and their Littlewood–Paley sums in the AMLR wavelet scattering network. For the ABR wavelet scattering network, the signal length was set to 450, the invariance scale was set to 0.006 s, and the sampling frequency was set to 30,000 Hz. For the AMLR wavelet scattering network, the signal length was set to 420, the invariance scale was set to 0.081 s, and the sampling frequency was set to 3000 Hz. 

Each WST was performed on the zeroth, first, and second orders after inputting the corresponding wavelet scattering network, and the wavelet scattering coefficients were outputted stepwise. For each signal, the zeroth-order scattering coefficients constituted the convolution of the original signal and the scale function; the first- and second-order scattering outputs constituted a two-dimensional matrix (scattering path x time window) including wavelet scattering coefficients. Based on the constructed scattering networks and their parameters, the AEP signals were presented to the wavelet scattering networks for the wavelet scattering transformations.

The extracted scattering features for ABR signals were 40 × 15 × 496. Each page of the tensor (40 × 15) was the scattering transform of one signal. The WST was critically down-sampled in time based on the bandwidth of the scaling function [73]. In this case, this resulted in 15 time windows for each of the 40 scattering paths. Respectively, the extracted scattering features for AMLR signals were 65 × 7 × 496. In this case, this resulted in 7 time windows for each of the 65 scattering paths.

#### 3.2.2. Dimensionality Reduction in WST

In order to obtain two matrices compatible with the available data frames, i.e., to match the generated wavelet coefficients with the ear from which they were derived, the two tensors had to be transformed appropriately. Specifically, wavelet scale averaging [77] was used for transforming the two-dimensional feature matrix of each signal into a one-dimensional feature vector to meet the input requirements for the classification model building. This method was averaged over the time window (wavelet scale) dimensions and resulted in a vector of averaged coefficients. Therefore, after the application of this method, 40 coefficients were obtained for each ABR signal and 65 coefficients for each AMLR signal. This led to a vector of coefficients for each AEP signal.

The vectors of the scattering coefficients were calculated and extracted using MATLAB. Then, three datasets were created with wavelet scattering coefficients as features. The first one included the ABR WST coefficients, the second one included the AMLR WST coefficients, and the third dataset included the WST coefficients of both AEPs (Table 3).

### 3.3. Patients’ Clinical Data Integration 

Following all the steps described in Section 2.6, a dataset resulted based on the UNITI project data. In the generated dataset, every row was about a different patient, and each column was about a different feature. This dataset contained 248 patients and 33 patients’ characteristics, used as features for our modelling. Table S1 describes in detail the variables that resulted for this dataset, as well as their description and range values.

### 3.4. Classification Models

This section presents all the performance tables of the classification models developed within the study, followed by the ROC curves of the classifiers with the highest AUC values among the models. Specifically, the tables present the results of the selected measures (AUC, sensitivity, and specificity) that quantified the discriminative accuracy of the seven classifiers in order to compare and identify the classifier with the highest classification performance for each dataset examined.

#### 3.4.1. Time-Domain Models

**Figure 9:**
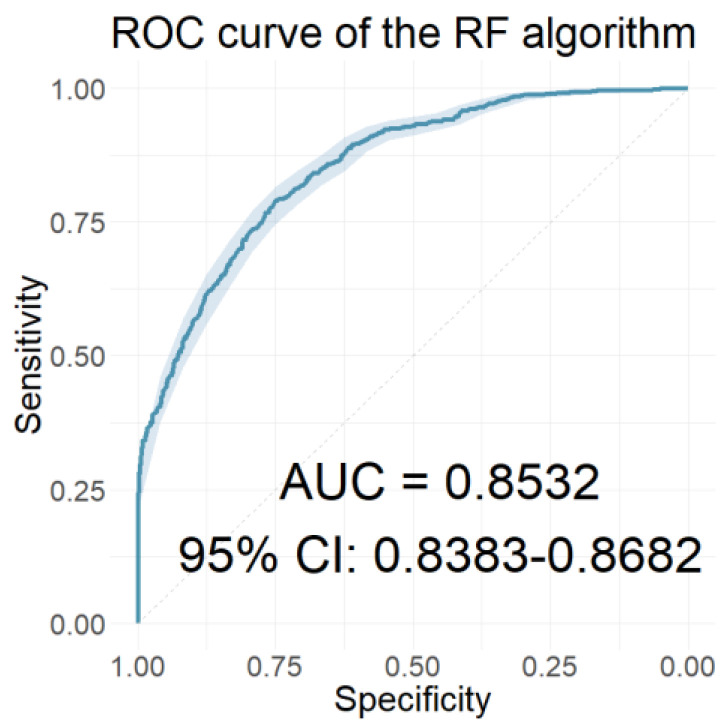
The ROC curve of the classifier using the 11 selected AEP metrics in time domain as features.

From the statistical analyses performed, statistically significant differences (*p*-value < 0.05) were found in the aforementioned metrics between the compared groups. These metrics were used as input features in the relevant classification models. Table 4 presents the resulting input features for our models. Table 5 shows how well each of these classification models performed, and Figure 9 shows the ROC curve of the RF classifier, which had the highest AUC value.

#### 3.4.2. Time–Frequency-Domain Models

**Figure 10:**
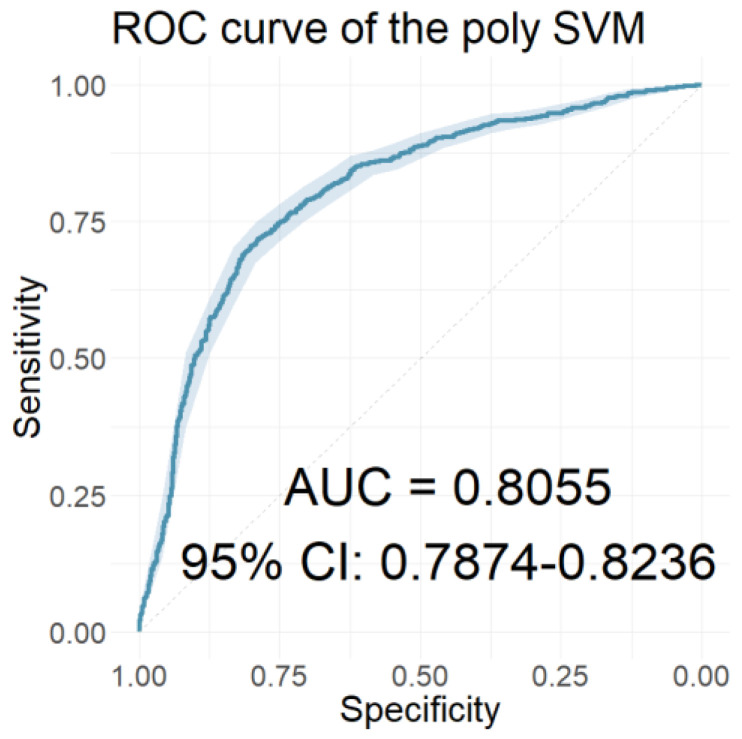
The ROC curve of the classifier with the highest AUC value for the models using the ABR WST coefficients as features.

**Figure 11:**
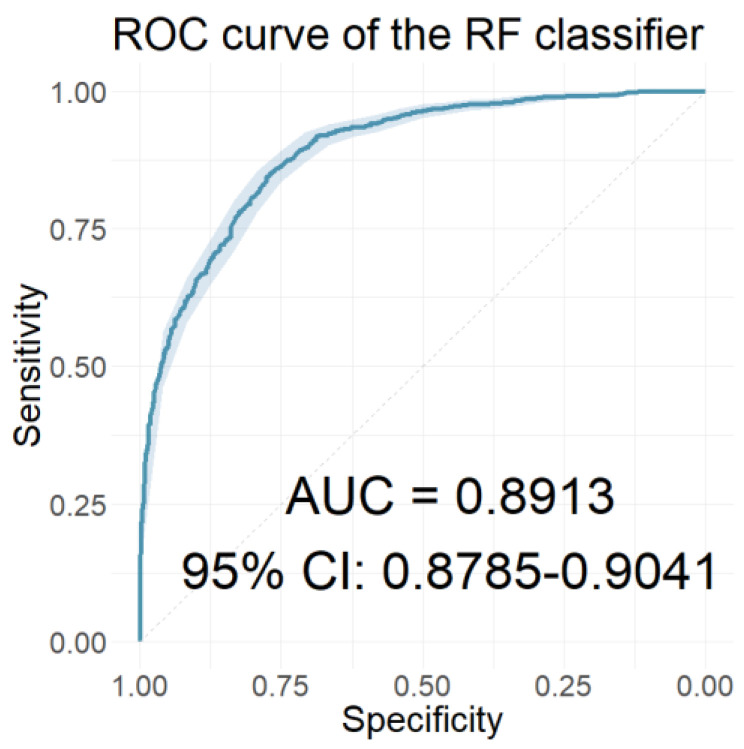
The ROC curve of the classifier with the highest AUC value for the models using the AMLR WST coefficients as features.

**Figure 12:**
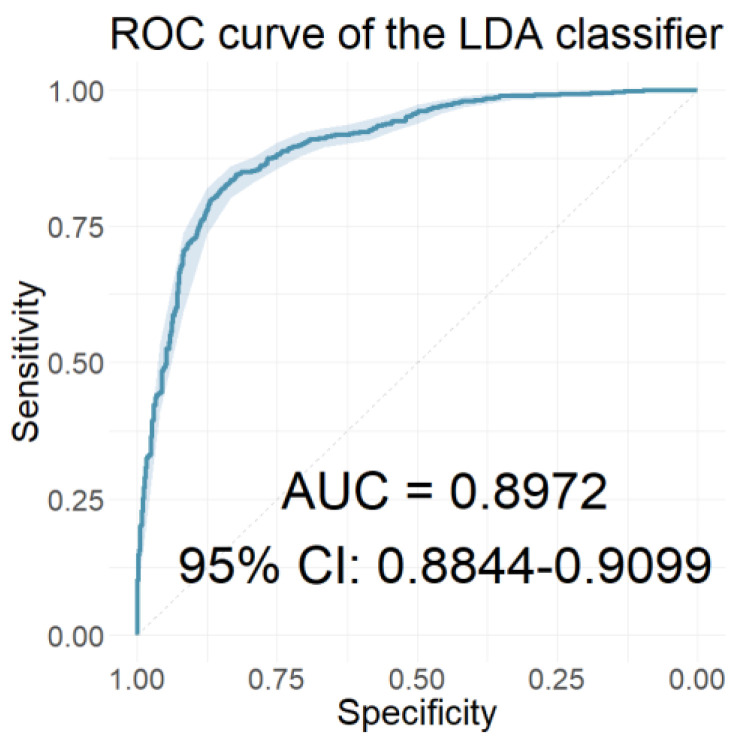
The ROC curve of the classifier with the highest AUC value for the models using both AEP subtypes’ WST coefficients as features.

In this section, we present the performance of the models developed using the resulting coefficients of the two wavelet scattering networks as input features. Table 6 presents the performances of the models with the 40 coefficients derived from the WST of the ABR signals as features. Table 7 presents the performances of the models with features of the 65 coefficients obtained from the WST of the AMLR signals. Finally, Table 8 presents the performances of the models that used all 105 coefficients from the WST for both AEP signals cumulatively. Similarly, Figure 10, Figure 11 and Figure 12 depict the ROC curves of the classifiers with the highest AUC value for each case.

#### 3.4.3. Integration of Clinical Features

**Figure 13:**
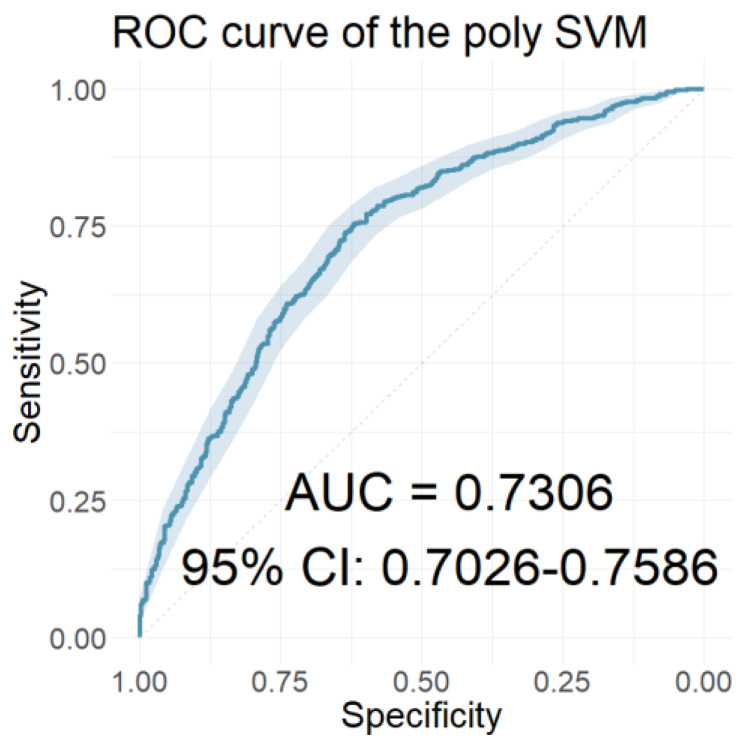
The ROC curve of the classifier with the highest AUC value for the models using all 33 clinical characteristics as features.

The performance of the classification models developed with all 33 clinical features is presented in Table 9. The ROC curve of the polynomial classifier, which had the highest AUC value, is illustrated in Figure 13.

**Figure 14:**
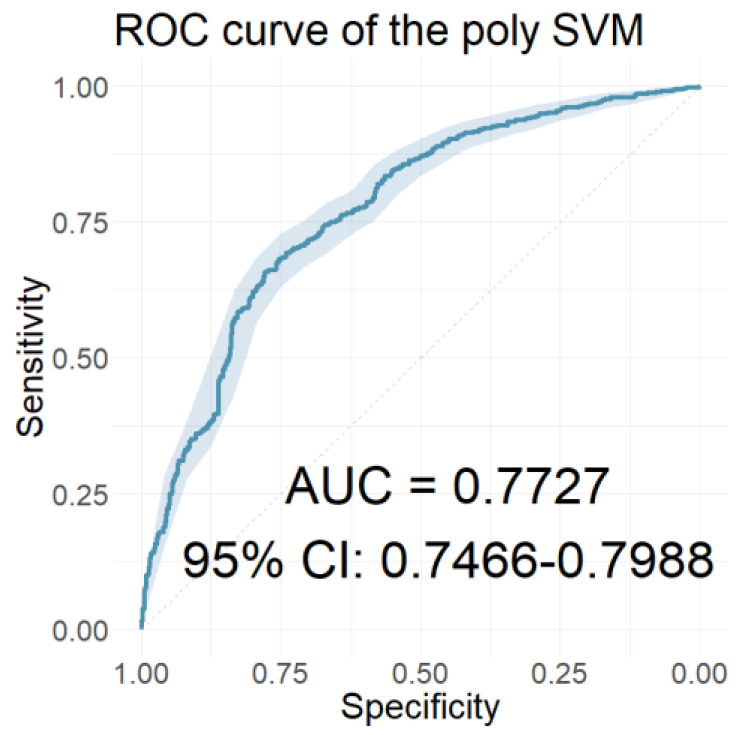
The ROC curve of the classifier with the highest AUC value for the models using the 15 LASSO-selected clinical characteristics as features.

Applying the LASSO regression, 15 out of 33 clinical data were selected as the most relevant. The names of the selected characteristics, the descriptions of which are included in Table S1, were the following: “Age”, “Height”, “Alcohol”, “Hearing loss 6000”, “Hearing loss”, “Matching type”, “Family history”, “Education”, “Vertigo”, “Frequency”, “Day pattern”, “Number sounds”, “Quality”, “Rhythmic”, and “GUF”. The classification models were developed using these 15 selected features. Their performances are displayed in Table 10. As can be seen from the table, all classification models performed better with the LASSO-selected subset of features. The ROC curve of the polynomial SVM classifier, which scored the highest AUC value, is illustrated in Figure 14. 

#### 3.4.4. Integrated Models Combining AEP and Clinical Features

**Figure 15:**
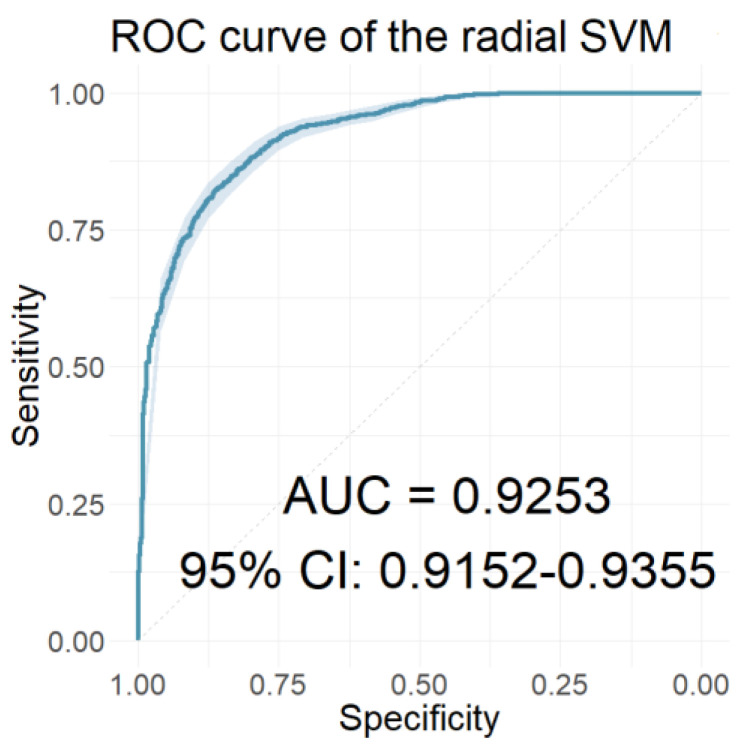
The ROC curve of the classifier with the highest AUC value for the models using the AMLR WST coefficients in combination with the 15 LASSO-selected clinical characteristics as features.

Observing the performances of all the developed models presented until this step, it appeared that models which used the resulting wavelet scattering coefficients of AMLR signals as input features scored the highest values for AUC, sensitivity, and specificity. To generate more accurate classification models using the same classifiers, we incorporated, along with the AMLR wavelet scattering coefficients (*n* = 65), the 15 selected clinical features as indicated by LASSO. Table 11 shows how well each of these classification models performed, and Figure 15 shows the ROC curve of the radial SVM classifier, which had the highest AUC value.

## 4. Discussion

The presence of tinnitus can profoundly affect thoughts, emotions, and dispositions. Its heterogeneity among patients results in a range of distress levels, from mild discomfort to the complete disruption of personal and professional life [2]. Objective measurements of tinnitus distress lag behind, with limited utilisation of evidence-based data and objective practices in the medical community. The severity classification currently relies on clinical guidelines, considering the occupational and social impacts [99]. This is conducted through graded self-report questionnaires [100] and structured medical histories [101], which are used for assessing tinnitus severity, distress, and quality of life impact.

The lack of tailored treatments for subjective tinnitus stems from its heterogeneity, impeding effective management for specific patient groups. Establishing an objective and reliable approach to detect and classify tinnitus distress would assist personalised treatment, reduce ENT clinic visits, lower healthcare costs, and enhance patient confidence and satisfaction.

Subjective tinnitus is attributed to aberrant neuronal activity in the auditory cortex, arising from disruptions or modifications in the auditory pathway [102]. This disturbance may lead to a loss of cortical activity suppression and the formation of new neural connections. Conductive hearing loss, caused by factors such as cerumen impaction or otitis media, can also be associated with subjective tinnitus by altering the sound input to the central auditory system. AEPs, such as the ABR waveforms paired with AMLR, offer an affordable and non-invasive means to examine the auditory pathway [103], providing valuable insights that may be beneficial for assessing the tinnitus pathophysiology.

In the present study, we aimed to extract features from both ABR and AMLR signals, as well as other clinical characteristics, and assess their performance in terms of distinguishing between low- and high-tinnitus-distress patient groups. In order to achieve this, we gathered several metrics from AEP signals and used them to build machine-learning models. In particular, we analysed conventional time-domain metrics, for which statistically significant differences were found between the two groups. Motivated by the effective application of the WST in EEG classification problems, we utilised this advanced transform method to extract coefficients as features in the time–frequency domain from the induced ABR and AMLR signals. To the best of our knowledge, this feature extraction method has not yet been applied to AEP signals. In an effort to conduct more in-depth research and contribute to the better profiling of bothersome tinnitus patients, we integrated their clinical characteristics, as determined by audiological examinations and questionnaire responses. After the selection of the most relevant clinical features, these were combined with the AEPs features, and integrated classification models were developed. Several well-known classifiers, including linear, radial, and poly SVM, RF, NB, NN, and LDA, were applied to all the generated datasets in order to evaluate the performance of the selected features in terms of predicting the level of discomfort caused by tinnitus.

The statistical analyses that were conducted using the THI score as a unique criterion for grouping participants revealed statistically significant differences in certain waves’ metrics. However, these differences were accompanied by effect sizes that ranged from small to moderate. The comparisons showing statistically significant differences were used as input features for the classification models. Specifically, the latencies of peaks III (*p* < 0.001) and V (*p* < 0.05) as well as the amplitudes of peaks I (*p* < 0.001) and V (*p* < 0.05) from the ABR signals, and the latencies of peaks Pa (*p* < 0.05) and Pb (*p* < 0.05) and trough Nb (*p* < 0.05), as well as the amplitudes of troughs Na (*p* < 0.001) and Nb (*p* < 0.05), and peaks Pa (*p* < 0.001) and Pb (*p* < 0.05) from the AMLR signals, were selected as features.

Comparing people of the same gender with the same hearing threshold level also revealed statistically significant differences in some of the metrics analysed. However, the number of significant differences decreased when comparing subgroups within each AEP subtype. Nevertheless, these comparisons showed moderate to large effect sizes, indicating stronger differences when applying stricter classification criteria. This suggests that a more homogeneous sample reduces extraneous factors that could dilute the effect size, resulting in a clearer relationship between variables. It is important to ensure that stricter classification remains relevant and unbiased. Statistical analyses revealed that alternations in auditory generators of several waves of interest are linked with tinnitus distress. Consequently, a greater or smaller amplitude and prolonged or shorter latency may indicate a problem with the synchronised activity of these generators [104]. Moreover, various neuro-physiological models of tinnitus perception may explain the variation in AEPs’ morphology and metrics among tinnitus sufferers. Neural synchrony appears to be a possible cause of tinnitus perception. However, due to differences in participant recruitment with respect to their tinnitus characteristics, sex, ageing, pharmacological status, and hearing loss, as well as discrepancies in the stimulus and acquisition parameters of the waveforms [105], divergent and contradictory results may arise when analysing AEPs in tinnitus sufferers. Our findings indicate that AEPs may be incorporated into the overall evaluation of tinnitus patients. However, before particular AEPs’ waves of interest can be recommended as biomarkers of tinnitus distress, additional in-depth research with a greater number of patients is necessary. To reach authoritative conclusions on the use of AEPs as a potential clinical diagnostic tool for tinnitus, more stringent criteria for patient categorization, including age range considerations, should be implemented. Consensus among clinicians regarding stimulus and acquisition parameters for recording these AEPs is equally important.

In the time domain, the classification models were built utilising the latencies and amplitudes of AEPs as their features, showcasing encouraging performance outcomes. Specifically, classification models were constructed, employing 11-waveform time-domain metrics with a *p*-value below 0.05. Among these models, the RF classifier achieved the highest AUC value of 0.8532, a sensitivity of 0.8097, and a specificity of 0.7005. 

In the time-frequency domain, the classification models were developed using the resulting coefficients of the two AEP subtypes’ wavelet scattering networks as input features. Initially, models that included only the wavelet scattering coefficients of one AEP subtype were developed. Comparing the performances of these models, the models using AMLR WST coefficients as features significantly outperformed those using ABR WST coefficients on all three performance measures in all seven classifiers. The RF classifier of the AMLR WST features performed best, with an AUC value of 0.8913, a sensitivity of 0.8192, and a specificity of 0.8101. Further progression involved developing models that incorporated scattering coefficients from both AEP subtypes. Of these models, the LDA classifier achieved optimal performance, with an AUC value, sensitivity, and specificity of 0.8972, 0.8486, and 0.8161, respectively. However, the rest of the models using the combined scattering coefficients also proved to be equally reliable, scoring correspondingly high values in both sensitivity and specificity. 

One noteworthy observation of the time-frequency domain models’ performances pertained to the close proximity of the AUC values in models that utilised AMLR WST coefficients as features compared to models incorporating WST coefficients of both AEP subtypes (consisting of 105 features). Notably, the AUC value of the radial SVM classifier in the AMLR model outperformed the corresponding classifier in the combined coefficient models. The similarity in AUC values across all cases suggests a stronger influence of AMLR waveforms in categorising the level of tinnitus discomfort compared to ABR signals. These findings are indirectly supported by the results of the statistical analyses, which revealed more statistically significant differences in AMLR than ABR metrics, particularly in cases where categorization relied solely on the THI score.

Across both the time- and the time–frequency-domain models exclusively utilising the ABR WST features, an intriguing pattern emerged: the specificity values consistently trailed behind the sensitivity values, irrespective of the employed classifier. This observation indicates potential challenges faced by these models in accurately identifying subjects with low distress (true negatives), consequently resulting in a heightened risk of false positives. These false positives occur when subjects with low distress are mistakenly classified as having high distress. In stark contrast to the aforementioned models, the models that incorporated the AMLR WST features, either independently or in conjunction with the ABR WST features, demonstrated remarkable performance across all three evaluation metrics: AUC, sensitivity, and specificity. These compelling findings unequivocally showcase the latter models’ exceptional proficiency in detecting and precisely classifying instances from both examined categories. Moreover, these results highlight the AMLR WST coefficients as the most informative features among the two AEP subtypes for the ongoing classification task.

In an effort to increase the performance of our classification models, we integrated patients’ clinical characteristics into our analysis, derived from UNITI’s project database. The relatively poor performances of these models, particularly in terms of specificity values, suggested the need for additional and more informative data for tinnitus distress classification. The better performances of the models, using the ABR and AMLR features, suggest that AEP signals will prove to be highly informative for tinnitus distress.

In order to create more robust classifiers, models that included the AMLR WST coefficients as features, combined with the 15 LASSO-selected clinical features, were developed. These integrated models achieved the highest classification performance, which indicates that the combination of multiple measures as features is the best choice for classification purposes. 

As was expected, the use of AEP signals to investigate tinnitus distress has some limitations. Tinnitus is subjective and varies greatly among individuals, posing challenges to accurately quantifying it with AEPs. Furthermore, AEPs provide limited insights into the underlying mechanisms of tinnitus and overlook the psychological and emotional factors contributing to the condition. Signal variability, background noise, and individual differences further constrain AEP analysis. To address these limitations, a comprehensive assessment can be achieved by combining multiple measures such as self-report questionnaires, behavioural assessments, and physiological measurements. Efforts towards standardisation, noise reduction, and tailored approaches are also essential for improving tinnitus evaluation. 

Our classification models faced additional limitations that influenced the outcomes. A major limitation was relying on THI questionnaire scores used to categorise sufferers. While self-reported measures are commonly utilised, they introduce bias, affecting judgements and responses [19]. Considering the diverse nature and varying severity of tinnitus, it is crucial for clinicians and researchers to ensure that participants have understood instructions so that they can respond accurately. Although qualified professionals supervised the questionnaires’ completion, precise classification based on scores proved challenging. Additionally, the absence of AEP signals from a control group limited our study further. Including both tinnitus and non-tinnitus signals would have enhanced our understanding of patient differences and allowed for more robust classification models. The latter was something we were unable to explore through the current study.

In conclusion, in this research we investigated the potential of ABR and AMLR signals for classifying ears into low and high distress groups. This study demonstrated that AMRL signals’ scattering coefficients may be efficiently employed for classification tasks, as they yielded excellent AUC values when implemented within the appropriate classifiers. According to our findings, the scattering coefficients derived from the averaged time windows in conjunction with the RF classifier yielded the higher performance. Regarding the interpretation of these results, the AMLR generators were structures along the thalamocortical pathway; however, particular sites are contested [103]. The rostral location of these AMLR generators relative to the ABR generator locations enabled the AMLR to provide insights into the operation of an additional part of the higher auditory system. In accordance with a previous study [106], individuals with tinnitus seemed to have more abnormalities in components of the AMLR when compared to individuals without tinnitus, suggesting alterations in the generation and transmission of neuroelectrical impulses along the auditory pathway. According to our results, it seems that there are differences in AMLR waveforms between sufferers with severe to catastrophic tinnitus distress and those with mild to moderate distress. These results may suggest that tinnitus is not related only to the higher brain areas.

## 5. Conclusions

Overall, several models were developed in this study in order to classify individuals with tinnitus based on the level of distress they experienced. Observing the models’ performance, and since no such study has been carried out before, we strongly encourage researchers to further investigate the potential use of the WST method for the classification of AEP signals in future studies. One proposal could be to create AEPs wavelet scattering networks by testing different values in their parameters (i.e., the size of the invariance scale, the number of filter banks, and the number of wavelets per octave in each filter bank), which may possibly lead to even higher classification yields. The classification is proposed to be related to both the level of tinnitus severity or discomfort and the presence or absence of tinnitus. Based on the results of our study, the utilisation and analysis of AMLR waveforms through the generated scattering coefficients scored remarkable AUC values. This suggests that AMLR signals could provide insightful information about tinnitus distress. Future investigations are therefore deemed more than necessary to validate the kinds of conclusions drawn from this study. 

The study’s added value lies in the development of standardised procedures for profiling patients with subjective tinnitus in a more accurate and meaningful manner. By identifying the most affected and distressed individuals, researchers and clinicians can investigate the crucial features that contribute to the classification of tinnitus sufferers in terms of distress. This research has the potential to enhance the assessment and management of subjective tinnitus, bringing benefits such as uniformity in treatment for adult patients, the utilisation of ongoing research results, the application of sophisticated data analysis tools, and advancements in personalised treatment and systems-based care. Furthermore, these findings have the capacity to contribute to the field’s knowledge, leading to more accurate and effective patient profiling, which could result in better treatment approaches, improved patient outcomes, and a heightened awareness of tinnitus management challenges.

## Figures and Tables

**Table 1:** Inclusion and exclusion criteria of the UNITI’s project RCT.

**Inclusion Criteria**
Tinnitus as primary complaint;
Chronic tinnitus (for at least 6 months based on history);
Age 18–80 years;
Ability to understand and consent to the research requirements, and to participate (hearing ability, intellectual capacity, no plans for sabbaticals or long-term holidays, no plans for pregnancy);
A score of >22 on the Montreal Cognitive Assessment (MoCA), i.e., adults without mild cognitive impairment [42];
Ability and willingness to use the UNITI mobile applications on their smartphones;
A score of ≥18 in the Tinnitus Handicap Inventory (THI) [43];
Willing to use a hearing aid (if there was indication);
If a drug therapy with psychoactive substances (e.g., antidepressants, anticonvulsants) existed at the beginning of the therapeutic intervention, it must have been stable for at least 30 days. The therapy should remain constant during the whole study, but a necessary change was not an exclusion criterion. Any change in medication was documented in the case report form (CRF).
**Exclusion Criteria**
Objective tinnitus/heartbeat-synchronous tinnitus as primary complaint;
Start of any other tinnitus-related treatments, especially hearing aids (HA), structured counselling, sound therapy (with special devices, expecting long term effects) or cognitive behavioural therapy in the last 3 months before the start of the study *;
Otosclerosis/acoustic neuroma or other relevant ear disorders with fluctuating hearing;
Present acute infections (e.g., acute otitis media, otitis externa, acute sinusitis);
Meniere’s disease or similar syndromes (but not vestibular migraine);
Serious internal, neurological, or psychiatric conditions;
Epilepsy or other CNS disorders (e.g., brain tumour, encephalitis);
Clinically relevant drug, medication or alcohol abuse up to 12 weeks before the start of the study;
Missing written informed consent;
Severe hearing loss—inability to communicate properly in the course of the study; 70 dB hearing level at 4 kHz (deviations were possible if there was a clinical justification for it);
One deaf ear.

* If an HA has already been worn for three months before screening, eligible candidates were allowed to participate but were automatically assigned to the no HA indication group.

**Table 2:** Stimulus and acquisition parameters for ABR and AMLR recordings.

**ABR**			
**Stimulus Parameters**		**Acquisition Parameters**	
Type of transducer	Insert phone	Analysis time	15 ms
Sample rate	30 kHz	Sweeps	4000
Type of stimulus	Click	Mode	Monaural
Polarity	Alternate	Electrode montage	Vertical (Fpz, Cz, M1/M2)
Repetition rate	Stimuli per second: 22 Hz	Filter setting for input amplifier	Low Pass: 1500 Hz; high Pass: 33 Hz, 6 dB per octave
Intensity	80 dB nHL	Preliminary display settings	Low pass: 1500 Hz;high Pass: 150 Hz
Masking	Off		
**AMLR**			
**Stimulus Parameters**		**Acquisition Parameters**	
Type of transducer	Insert phone	Analysis time	150 ms
Sample rate	3 kHz	Sweeps	500
Type of stimulus	2 kHz Tone Burst, Manual window	Mode	Monaural
Duration of stimulus	total of 28 sine waves; rise/fall: 4; plateau: 20	Electrode montage	Vertical (Fpz, Cz, M1/M2)
Polarity	Rarefaction	Filter setting for input amplifier	Low Pass: 1500 Hz; high Pass: 10 Hz, 12 dB per octave
Repetition rate	Stimuli per second: 6.1 Hz	Preliminary display settings	Low pass: 100 Hz; high Pass: 15 Hz
Intensity	70 dB nHL		
Masking	Off		

**Table 3:** WST dataset variables.

Number of Features	Description	Type of Values
40	ABR scattering coefficients	Numeric
65	AMLR scattering coefficients	Numeric
105	ABR and AMLR scattering coefficients	Numeric

**Table 4:** Selected AEP metric features in time domain.

	Description	Range of Values
1	III peak latency	Numeric
2	V peak latency	Numeric
3	I peak amplitude	Numeric
4	V peak amplitude	Numeric
5	Pa peak latency	Numeric
6	Nb trough latency	Numeric
7	Pb peak latency	Numeric
8	Na trough amplitude	Numeric
9	Pa peak amplitude	Numeric
10	Nb trough amplitude	Numeric
11	Pb peak amplitude	Numeric

**Table 5:** Performance results of the classification models using the 11 selected AEP metrics in time domain as features. Bold: the one with the highest AUC value.

Machine Learning Classifier	No of Features	AUC	Sensitivity	Specificity
LDA	11	0.7213	0.7879	0.5411
Linear SVM	11	0.7131	0.7895	0.5171
NB	11	0.7482	0.7394	0.6410
NN	11	0.7300	0.7220	0.6578
Poly SVM	11	0.7462	0.8359	0.5516
Radial SVM	11	0.7667	0.7762	0.6619
**RF**	**11**	**0.8532**	**0.8097**	**0.7005**

**Table 6:** Performance results of the classification models using the ABR WST coefficients as features. Bold: the one with the highest AUC value.

Machine-Learning Classifier	No. of Features	AUC	Sensitivity	Specificity
LDA	40	0.7970	0.7743	0.6639
Linear SVM	40	0.8023	0.7897	0.6322
NB	40	0.6522	0.4767	0.7234
NN	40	0.7986	0.7609	0.6796
**Poly SVM**	**40**	**0.8055**	**0.8302**	**0.6729**
Radial SVM	40	0.7331	0.7498	0.5858
RF	40	0.7923	0.7762	0.6819

**Table 7:** Performance results of the classification models using the AMLR WST coefficients as features. Bold: the one with the highest AUC value.

Machine-Learning Classifier	No. of Features	AUC	Sensitivity	Specificity
LDA	65	0.8715	0.8009	0.7849
Linear SVM	65	0.8459	0.8122	0.7374
NB	65	0.7464	0.6560	0.7450
NN	65	0.8595	0.8167	0.7722
Poly SVM	65	0.8816	0.8335	0.7711
Radial SVM	65	0.8836	0.8257	0.7618
**RF**	**65**	**0.8913**	**0.8192**	**0.8101**

**Table 8:** Performance results of the classification models using both AEP subtypes’ WST coefficients as features. Bold: the one with the highest AUC value.

Machine-Learning Classifier	No. of Features	AUC	Sensitivity	Specificity
**LDA**	**105**	**0.8972**	**0.8486**	**0.8161**
Linear SVM	105	0.8902	0.8622	0.8040
NB	105	0.7441	0.6293	0.7488
NN	105	0.8926	0.8295	0.8158
Poly SVM	105	0.8966	0.8526	0.7934
Radial SVM	105	0.8703	0.8234	0.7418
RF	105	0.8903	0.8260	0.7887

**Table 9:** Performance results of the classification models using all 33 clinical characteristics as features. Bold: the one with the highest AUC value.

Machine-Learning Classifier	No. of Features	AUC	Sensitivity	Specificity
LDA	33	0.6981	0.7310	0.5495
Linear SVM	33	0.6834	0.7789	0.4508
NB	33	0.6928	0.7771	0.4466
NN	33	0.5368	0.7256	0.3154
**Poly SVM**	**33**	**0.7306**	**0.7496**	**0.5667**
Radial SVM	33	0.7221	0.7116	0.6150
RF	33	0.7034	0.7157	0.5612

**Table 10:** Performance results of the classification models using the 15 LASSO-selected clinical characteristics as features. Bold: the one with the highest AUC value.

Machine-Learning Classifier	No. of Features	AUC	Sensitivity	Specificity
LDA	15	0.7560	0.7460	0.6374
Linear SVM	15	0.7649	0.7819	0.6159
NB	15	0.7548	0.7792	0.5662
NN	15	0.7390	0.5929	0.7342
**Poly SVM**	**15**	**0.7727**	**0.7732**	**0.6680**
Radial SVM	15	0.7493	0.7392	0.6261
RF	15	0.7186	0.7505	0.5867

**Table 11:** Performance results of the classification models using the AMLR WST coefficients in combination with the 15 LASSO-selected clinical characteristics as features. Bold: the one with the highest AUC value.

Machine-Learning Classifier	No. of Features	AUC	Sensitivity	Specificity
LDA	80	0.8886	0.8128	0.7690
Linear SVM	80	0.9087	0.8591	0.8201
NB	80	0.8098	0.7053	0.7981
NN	80	0.9075	0.8367	0.8395
Poly SVM	80	0.9250	0.8647	0.8599
**Radial SVM**	**80**	**0.9253**	**0.8484**	**0.8304**
RF	80	0.9240	0.8598	0.8209

## Data Availability

The data presented in this study are available on reasonable request from the corresponding author.

## Supplementary Materials

The following supporting information can be downloaded at: https://www.mdpi.com/article/10.3390/jcm12113843/s1, title; Figure S1. The QQ plot was used to test whether the dependent variable “Pb latency” followed a normal distribution; Figure S2. Boxplots of ABR waveform latencies based on tinnitus distress in people with common hearing levels and gender; Figure S3. Boxplots of ABR waveform amplitudes based on tinnitus distress in people with common hearing levels and gender; Figure S4. Boxplots of AMLR waveform latencies based on tinnitus distress in people with common hearing levels and gender; Figure S5. Boxplots of AMLR waveform amplitudes based on tinnitus distress in people with common hearing levels and gender; Table S1: The 33 Selected Clinical Variables; Table S2: Descriptive statistics regarding the tinnitus distress influence on ABR components; Table S3: Descriptive statistics regarding the tinnitus distress influence on AMLR components; Table S4: Statistical differences regarding the tinnitus distress influence on ABR components; Table S5: Statistical differences regarding the tinnitus distress influence on AMLR components; Table S6: Descriptive statistics regarding the level of tinnitus distress on the components of the ABR waveforms in people with common hearing levels and gender; Table S7: Descriptive statistics regarding the level of tinnitus distress on the components of the AMLR waveforms in people with common hearing levels and gender; Table S8: Statistical analyses regarding the level of tinnitus distress on the components of the ABR waveforms in people with common hearing levels and gender; Table S9: Statistical analyses regarding the level of tinnitus distress on the components of the AMLR waveforms in people with common hearing levels and gender.
