# Supplementary material for: Development of Machine-Learning Models for Tinnitus-Related Distress Classification Using Wavelet-Transformed Auditory Evoked Potential Signals and Clinical Data
Source: J Clin Med. 2023 Jun 4;12(11):3843. doi: 10.3390/jcm12113843 (PMC10253417; doi:10.3390/jcm12113843)
Supplement: Supplementary file 1 [file jcm-12-03843-s001.zip › jcm-2232831-supplementary.pdf]

Table S1. The 33 Selected Clinical variables.

	Name	Description	Range of Values
1	Age	Individual's age	Numeric
2	Height	Individual's height	Numeric
3	Weight	Individual's weight	Numeric
4	Alcohol	The average number of alcoholic drinks that the individual consumes per week (1 drink = 125 ml glass of wine, 330 ml of beer, 40 ml of spirits)	Numeric
5	Tinnitus time	The number of months that the individual has experienced tinnitus	Numeric
6	Tinnitus bother time	The length of time the individual has been bothered by tinnitus (in months)	Numeric
7	Hearing loss 250	Ear's hearing loss at 250 Hz	Numeric
8	Hearing loss 500	Ear's hearing loss at 500 Hz	Numeric
9	Hearing loss 1000	Ear's hearing loss at 1 kHz	Numeric
10	Hearing loss 2000	Ear's hearing loss at 2 kHz	Numeric
11	Hearing loss 3000	Ear's hearing loss at 3 kHz	Numeric
12	Hearing loss 4000	Ear's hearing loss at 4 kHz	Numeric
13	Hearing loss 6000	Ear's hearing loss at 6 kHz	Numeric
14	Hearing loss 8000	Ear's hearing loss at 8 kHz	Numeric
15	Hearing loss	Ear’s hearing loss category	0 = Normal hearing (0-20 dB HL)<br>1 = Mild hearing loss (21-60 dB HL)<br>2 = Severe hearing loss (61-x dB HL)
16	Gender	Individual's gender	1 = Female<br>2 = Male
17	Family history	Existence of a first-degree relative with tinnitus or hearing loss (parents, children, siblings)	0 = No<br>1 = Yes
18	Education	The highest education level of the individual	1 = No school<br>2 = Primary (elementary school)<br>3 = Lower secondary (middle school)<br>4 = Upper secondary (high school)<br>5 = University or higher degree
19	Smoking	Individual's smoking status	1 = Never smoker<br>2 = Current smoker<br>3 = Ex-smoker
20	Vertigo	Individual's answer to whether they suffer from vertigo (sensation of spinning or tilting)	1 = Never<br>2 = Yes, at least one episode per year<br>3 = Yes, less than one episode per year
21	Frequency	Individual's answer to how often they have tinnitus on average	1 = Daily or almost daily<br>2 = Almost weekly<br>3 = Almost monthly<br>4 = Every few months<br>5 = Yearly
22	Day pattern	Individual's answer to what best describes their tinnitus during the day	1 = Constant<br>2 = Intermittent
23	Number sounds	Individual's answer to whether they hear one or more different sounds	1 = One sound<br>2 = More than one
24	Loudness changes	Individual's answer to whether the loudness of their tinnitus is stable over time or fluctuates over a day	1 = Stable<br>2 = Sometimes fluctuating<br>3 = Always fluctuating
25	Sound quality	Individual's answer to what their tinnitus sounds like	1 = Tonal<br>2 = Noise like<br>3 = Music like<br>4 = Cricket<br>5 = Other
26	Pitch	Tinnitus frequency	1= < 4000kHz<br>2= ≥ 4000kHz<br>3 = Combination of 1 and 2
27	Localisation	Individual's answer to where do they perceive their tinnitus	0 = Unilateral (non-tinnitus side)<br>1 = Unilateral (tinnitus side)

			2 = Bilateral<br>3 = Head
28	Rhythmic	Individual's answer to whether their tinnitus is rhythmic	1 = No<br>2 = Yes, following heat beat<br>3 = Yes, following breathing<br>4 = Yes following movements of the head, neck, jaw or muscles of the face<br>5 = Other
29	Matching type	Ear's tinnitus type	0 = Pure tone<br>1 = Narrow band<br>2 = Broad band
30	GUF	Score on the GUF questionnaire	Numeric (0–45)
31	Max Frequency	Expressing the ear's maximal tinnitus frequency	Numeric
32	Matching loudness	Expressing the ear's tinnitus matching loudness in dB	Numeric
33	Minimal masking	Expressing the ear's minimal masking level in dB	Numeric

Table S2. Descriptive statistics regarding the tinnitus distress influence on ABR components.

		THI score			THI score			THI score	
		≥48	<48		≥48	<48		≥48	<48
Waveforms		228	268		228	268		228	268
Mean	I latency	**1.48**	**1.54**	III latency	**3.62**	**3.72**	V latency	**5.46**	**5.59**
Median		1.45	1.52		3.58	3.68		5.38	5.55
SD		0.32	0.37		0.27	0.31		0.46	0.46
Minimum		0.7	0.72		2.9	2.92		4.52	4.52
Maximum		2.48	2.5		4.45	4.5		6.82	7
Mean	I amplitude	**0.1**	**0.07**	III amplitude	**0.21**	**0.19**	V amplitude	**0.21**	**0.19**
Median		0.09	0.06		0.21	0.18		0.21	0.18
SD		0.11	0.08		0.14	0.13		0.11	0.1
Minimum		-0.18	-0.18		-0.19	-0.12		-0.12	-0.12
Maximum		0.8	0.37		1.35	0.77		0.72	0.52

Table S3. Descriptive statistics regarding the tinnitus distress influence on AMLR components.

		THI score			THI score			THI score			THI score	
		≥48	<48		≥48	<48		≥48	<48		≥48	<48
Waveforms		228	268		228	268		228	268		228	268
Mean	Na latency	**19.54**	**18.77**	Pa latency	**29.33**	**27.98**	Nb latency	**41.03**	**39.7**	Pb latency	**50.51**	**48.76**
Median		18.84	18.83		28.17	27.5		40.17	39.16		50.17	48.83
SD		4.15	4.26		4.87	4.23		6.87	6.13		7.57	7.2
Minimum		10.5	10		20.17	18.33		27.17	26.67		31.84	31.83
Maximum		30	29.83		46	43.83		62.67	60.83		67.5	78.83
Mean	Na amplitude	**-0.45**	**-0,33**	Pa amplitude	**0.47**	**0.36**	Nb amplitude	**-0.4**	**-0.32**	Pb amplitude	**0.29**	**0.2**
Median		-0.4	-0.29		0.43	0.33		-0.39	-0.3		0.26	0.17
SD		0.34	0.3		0.29	0.25		0.28	0.25		0.31	0.27
Minimum		-1.77	-1.57		-0.33	-0.11		-1.39	-1.4		-0.45	-0.48
Maximum		0.21	0.44		1.72	1.32		0.54	0.63		1.78	1.21

Table S4. Statistical differences regarding the tinnitus distress influence on ABR components.

	Latency			Amplitude		
	**I**	**III**	**V**	**I**	**III**	**V**
Statistical Test	Welch's t-test	t-test	t-test	Welch's t-test	t-test	t-test
THI Score≥48 Vs Score<48	t(462.7) = -1.84, p = .066, 95% CI [-0.12, 0]	t(465) = -3.71, **p = <.001**, 95% CI [-0.15, -0.05]	t(491) = -3.09, **p = .002**, 95% CI [-0.21, -0.05].	t(368.26) = 3.64, **p = <.001**, 95% CI [0.02, 0.05]	t(465) = 1.73, p = .085, 95% CI [-0.01, 0.05]	t(491) = 2.55, **p = .011**, 95% CI [0.01, 0.04]
Size effect	d = 0.17	d = 0.34	d = 0.28	d = 0.34	d = 0.16	d = 0.23

Table S5. Statistical differences regarding the tinnitus distress influence on AMLR components.

	Latency				Amplitude			
	**Na**	**Pa**	**Nb**	**Pb**	**Na**	**Pa**	**Nb**	**Pb**
Statistical Test	t-test	t-test	t-test	t-test	t-test	t-test	t-test	t-test
THI Score≥48 Vs Score<48	t(448) = 1.93, p = .054, 95% CI [-0.02, 1.56]	t(452) = 3.17, **p = .002**, 95% CI [0.51, 2.2]	t(452) = 2.18, **p = .03**, 95% CI [0.12, 2.54]	t(452) = 2.51, **p = .012**, 95% CI [0.37, 3.12]	t(448) = -4.05, **p <.001**, 95% CI [-0.18, -0.06]	t(452) = 4.43, **p <.001**, 95% CI [0.06, 0.16]	t(452) = -3.26, **p = .001**, 95% CI [-0.13, -0.03]	t(452) = 3.06, **p = .002**, 95% CI [0.03, 0.14]
Size effect	d = 0.18	d = 0.3	d = 0.21	d = 0.24	d = 0.38	d = 0.42	d = 0.31	d = 0.29

Table S6. Descriptive statistics regarding the level of tinnitus distress on the components of the ABR waveforms in people with common hearing levels and gender.

		Normal Hearing				Mild Hearing Loss				Severe Hearing Loss			
		Females		Males		Females		Males		Females		Males	
		THI≥48	THI<48	THI≥48	THI<48	THI≥48	THI<48	THI≥48	THI<48	THI≥48	THI<48	THI≥48	THI<48
	Waveforms	22	26	24	32	76	56	67	109	12	10	27	35
**I latency**	**Mean**	**1.4**	**1.41**	**1.45**	**1.5**	**1.47**	**1.49**	**1.54**	**1.59**	**1.32**	**1.54**	**1.57**	**1.64**
	Median	1.38	1.44	1.45	1.55	1.48	1.48	1.52	1.57	1.42	1.42	1.58	1.58
	SD	0.18	0.25	0.31	0.33	0.23	0.29	0.43	0.42	0.35	0.57	0.32	0.36
	Min	1.02	0.75	0.75	0.72	0.85	0.85	0.75	0.72	0.7	0.88	1.02	0.98
	Max	1.68	1.82	2.25	2.32	2.45	2.32	2.48	2.5	1.78	2.45	2.25	2.5
**III latency**	**Mean**	**3.55**	**3.54**	**3.57**	**3.7**	**3.62**	**3.72**	**3.68**	**3.72**	**3.54**	**3.84**	**3.64**	**3.84**
	Median	3.52	3.53	3.57	3.65	3.58	3.68	3.67	3.72	3.47	3.78	3.62	3.82
	SD	0.21	0.17	0.2	0.2	0.24	0.28	0.31	0.35	0.28	0.26	0.33	0.33
	Min	3.22	3.22	3.08	3.35	3.25	2.98	3.02	2.92	3.08	3.45	2.9	3.12
	Max	4.02	4.05	4.08	4.35	4.38	4.5	4.45	4.48	4.02	4.32	4.28	4.48
**V latency**	**Mean**	**5.23**	**5.28**	**5.38**	**5.41**	**5.31**	**5.46**	**5.61**	**5.69**	**5.22**	**5.65**	**5.85**	**5.84**
	Median	5.27	5.3	5.35	5.48	5.28	5.45	5.48	5.58	5.28	5.57	5.75	5.72
	SD	0.29	0.34	0.26	0.36	0.33	0.35	0.5	0.46	0.48	0.29	0.58	0.58
	Min	4.78	4.52	4.88	4.58	4.68	4.52	4.52	4.68	4.55	5.28	4.95	4.62
	Max	5.82	5.87	6.08	6.18	6.35	6.35	6.58	6.95	5.98	6.15	6.82	7
**I amplitude**	**Mean**	**0.15**	**0.1**	**0.09**	**0.07**	**0.11**	**0.07**	**0.09**	**0.06**	**0.09**	**0.05**	**0.05**	**0.06**
	Median	0.16	0.09	0.1	0.06	0.09	0.07	0.09	0.06	0.1	0.05	0.05	0.06
	SD	0.11	0.11	0.12	0.07	0.14	0.09	0.08	0.07	0.08	0.07	0.08	0.07
	Min	-0.06	-0.13	-0.18	-0.14	-0.07	-0.18	-0.11	-0.15	-0.05	-0.05	-0.14	-0.07
	Max	0.46	0.37	0.33	0.2	0.8	0.27	0.42	0.33	0.21	0.14	0.21	0.25
**III amplitude**	**Mean**	**0.28**	**0.29**	**0.25**	**0.24**	**0.25**	**0.24**	**0.15**	**0.15**	**0.2**	**0.11**	**0.13**	**0.12**
	Median	0.28	0.29	0.23	0.23	0.24	0.22	0.14	0.14	0.22	0.13	0.09	0.08
	SD	0.09	0.1	0.08	0.12	0.17	0.11	0.1	0.11	0.08	0.11	0.12	0.11
	Min	0.12	0.1	0.14	-0.05	-0.19	-0.03	-0.07	-0.05	0.07	-0.12	-0.08	-0.06
	Max	0.45	0.49	0.37	0.52	1.35	0.52	0.41	0.77	0.32	0.27	0.33	0.39
**V amplitude**	**Mean**	**0.21**	**0.23**	**0.24**	**0.18**	**0.24**	**0.24**	**0.19**	**0.18**	**0.21**	**0.14**	**0.16**	**0.14**
	Median	0.22	0.21	0.23	0.17	0.24	0.24	0.19	0.16	0.2	0.13	0.15	0.12
	SD	0.09	0.11	0.1	0.11	0.13	0.09	0.08	0.09	0.06	0.13	0.09	0.1
	Min	0.03	0.02	0.06	-0.07	-0.12	0.08	0.02	-0.07	0.12	-0.04	-0.1	-0.12
	Max	0.39	0.46	0.52	0.43	0.72	0.45	0.41	0.52	0.31	0.33	0.31	0.47

Table S7. Descriptive statistics regarding the level of tinnitus distress on the components of the AMLR waveforms in people with common hearing levels and gender.

		Normal Hearing				Mild Hearing Loss				Severe Hearing Loss			
		Females		Males		Females		Males		Females		Males	
		THI≥48	THI<48	THI≥48	THI<48	THI≥48	THI<48	THI≥48	THI<48	THI≥48	THI<48	THI≥48	THI<48
	Waveforms	22	26	24	32	76	56	67	109	12	10	27	35
Na latency	**Mean**	**18.17**	**17.55**	**19.85**	**18.55**	**19.15**	**17.87**	**19.78**	**19.36**	**19.78**	**20.66**	**20.35**	**19.01**
	Median	17.84	17	18.5	18.5	18.67	17.83	19.67	19.83	20	19.66	21.17	18.66
	SD	3.63	3.27	3.76	4.24	3.88	4.02	4.26	4.62	4.6	3.07	4.91	4.15
	Min	10.84	11.83	14.5	10.83	10.5	10	10.5	10	10.5	17.83	10.84	11.83
	Max	25.17	26.16	29.5	27.16	27.84	27.5	28.5	29.16	26.84	27.16	30	29.83
Pa latency	**Mean**	**28.36**	**27.14**	**30.89**	**28.2**	**28.53**	**27.87**	**29.64**	**28.22**	**29.2**	**27.2**	**29.8**	**28.03**
	Median	27.67	27.16	28.17	28.83	27.84	27.16	28.84	27.83	29	27.16	29.5	27.5
	SD	3.01	3.54	7.04	3.98	4.91	3.88	4.44	4.56	3.47	3.47	4.97	4.74
	Min	23.5	18.33	23.17	19.5	20.5	22.16	20.84	18.83	24.17	20.5	20.17	18.83
	Max	34.84	34.33	46	38.5	44.84	39.83	40.84	43.83	35.5	32.5	40.84	39.16
Nb latency	**Mean**	**42.84**	**39.16**	**41.3**	**41.13**	**40.82**	**39.97**	**41.58**	**39.59**	**39.42**	**39.05**	**39.63**	**38.87**
	Median	42.67	37.83	38.84	39.83	40.5	39.75	40	39.5	37.5	38.16	40.17	39.16
	SD	6.22	4.79	8.88	6.94	6.54	6.98	6.71	5.92	6.5	5.14	6.87	5.94
	Min	29.84	31.5	31.84	26.67	27.17	26.83	28.5	26.67	32.17	32.16	27.84	28.5
	Max	51.84	50.67	62.67	55.16	59.17	60.83	58.84	56.16	53.17	49.16	54.84	51.16
Pb latency	**Mean**	**53.84**	**47.74**	**50.67**	**50.23**	**50.71**	**48.68**	**50.51**	**49.09**	**49.78**	**50.27**	**48.27**	**46.91**
	Median	56.17	47.5	49.5	49.83	50.84	47.5	49	49	47.67	48.83	48.17	45.5
	SD	7.25	4.83	7.92	7.41	7.15	9.26	7.96	6.38	7.07	6.62	7.58	7.49
	Min	39.84	41.5	40.84	37.5	35.17	31.83	31.84	31.83	42.84	41.83	36.84	32.16
	Max	64.5	56.67	65.84	64.83	66.5	78.83	67.5	63.16	62.84	64.5	62.17	61.16
Na amplitude	**Mean**	**-0.41**	**-0.43**	**-0.59**	**-0.45**	**-0.46**	**-0.39**	**-0.42**	**-0.26**	**-0.39**	**-0.15**	**-0.47**	**-0.31**
	Median	-0.39	-0.39	-0.41	-0.43	-0.38	-0.36	-0.37	-0.23	-0.41	-0.13	-0.43	-0.29
	SD	0.22	0.37	0.48	0.32	0.39	0.33	0.29	0.25	0.33	0.22	0.25	0.24
	Min	-0.76	-1.37	-1.7	-1.29	-1.77	-1.57	-1.13	-1.55	-0.94	-0.51	-0.99	-1.09
	Max	0.02	0.34	-0.1	0.01	0.05	0.2	0.17	0.44	0.21	0.16	0.01	0.31
Pa amplitude	**Mean**	**0.5**	**0.45**	**0.56**	**0.49**	**0.52**	**0.4**	**0.45**	**0.31**	**0.38**	**0.28**	**0.37**	**0.31**
	Median	0.46	0.45	0.45	0.43	0.49	0.36	0.43	0.3	0.42	0.31	0.38	0.28
	SD	0.24	0.21	0.39	0.28	0.33	0.3	0.26	0.22	0.32	0.14	0.13	0.2
	Min	0.11	0.04	0.06	0.07	-0.39	-0.11	-0.1	-0.07	-0.07	0.05	0.04	-0.07
	Max	1.05	0.89	1.72	1.18	1.56	1.32	1.11	1.28	0.91	0.56	0.6	0.78
Nb amplitude	**Mean**	**-0.41**	**-0.44**	**-0.39**	**-0.46**	**-0.48**	**-0.32**	**-0.37**	**-0.28**	**-0.44**	**-0.34**	**-0.3**	**-0.26**
	Median	-0.41	-0.45	-0.36	-0.4	-0.47	-0.29	-0.37	-0.27	-0.45	-0.35	-0.29	-0.25
	SD	0.28	0.22	0.24	0.3	0.31	0.23	0.24	0.24	0.38	0.13	0.25	0.22
	Min	-0.99	-1.15	-0.86	-1.4	-1.39	-1.02	-0.93	-1.19	-1.07	-0.54	-0.85	-0.95
	Max	0.06	-0.14	-0.02	-0.03	0.12	0.1	0.54	0.63	0.08	-0.13	0.3	0.18
Pb amplitude	**Mean**	**0.28**	**0.27**	**0.29**	**0.14**	**0.32**	**0.2**	**0.24**	**0.2**	**0.33**	**0.29**	**0.29**	**0.19**
	Median	0.27	0.22	0.26	0.13	0.24	0.21	0.23	0.15	0.4	0.25	0.28	0.18
	SD	0.19	0.39	0.3	0.27	0.35	0.28	0.31	0.25	0.35	0.19	0.23	0.22
	Min	-0.14	-0.45	-0.37	-0.4	-0.26	-0.48	-0.45	-0.34	-0.36	0.03	-0.24	-0.23
	Max	0.5	1.03	0.81	0.82	1.78	1.1	1.33	1.21	0.86	0.65	0.73	0.79

Table S8. Statistical analyses regarding the level of tinnitus distress on the components of the ABR waveforms in people with common hearing levels and gender.

	**Normal Hearing**						
		**Latencies (females)**			**latencies (males)**		
		**I**	**III**	**V**	**I**	**III**	**V**
t-test	THI Score≥48 Vs Score<48	t(45) = 0.15, p = .878, 95% CI [-0.12, 0.14]	t(46) = -0.04, p = .965, 95% CI [-0.11, 0.11]	t(46) = 0.58, p = .567, 95% CI [-0.13, 0.24]	t(52) = -0.52, p = .607, 95% CI [-0.23, 0.13]	t(53) = -2.38, **p = .021**, 95% CI [-0.24, -0.02]	t(53.94) = -0.29, p = .776, 95% CI [-0.19, 0.14]
	Statistical Test	t-test	t-test	t-test	t-test	t-test	Welch's t-test
	size effect	d = 0.05	d = 0.01	d = 0.17	d = 0.14	d = 0.65	d = 0.08
		**Amplitudes (females)**			**Amplitudes (males)**		
		**I**	**III**	**V**	**I**	**III**	**V**
t-test	THI Score≥48 Vs Score<48	t(45) = -1.4, p = .168, 95% CI [-0.11, 0.02]	t(46) = 0.49, p = .625, 95% CI [-0.04, 0.07]	t(46) = 0.61, p = .547, 95% CI [-0.04, 0.07]	t(52) = 1.01, p = .317, 95% CI [-0.03, 0.08]	t(50.54) = 0.39, p = .698, 95% CI [-0.04, 0.07]	t(54) = 2.24, **p = .029**, 95% CI [0.01, 0.12]
	Statistical Test	t-test	t-test	t-test	t-test	Welch's t-test	t-test
	size effect	d = 0.41	d = 0.14	d = 0.18	d = 0.28	d = 0.1	d = 0.6
	**Mild Hearing Loss**						
		**Latencies (females)**			**Latencies (males)**		
		**I**	**III**	**V**	**I**	**III**	**V**
t-test	THI Score≥48 Vs Score<48	t(124) = -0.3, p = .763, 95% CI [-0.11, 0.08]	t(128) = -2.23, **p = .028**, 95% CI [-0.19, -0.01]	t(129) = -2.45, **p = .016**, 95% CI [-0.26, -0.03]	t(160) = -0.78, p = .437, 95% CI [-0.19, 0.08]	t(157) = -0.78, p = .438, 95% CI [-0.15, 0.07]	t(172) = -1.15, p = .252, 95% CI [-0.23, 0.06]
	Statistical Test	t-test	t-test	t-test	t-test	t-test	t-test
	size effect	d = 0.05	d = 0.39	d = 0.43	d = 0.13	d = 0.13	d = 0.18
		**Amplitudes (females)**			**Amplitudes (males)**		
		**I**	**III**	**V**	**I**	**III**	**V**
t-test	THI Score≥48 Vs Score<48	t(116,49) = 2.22, **p = .028**, 95% CI [0, 0.09]	t(128) = 0.49, p = .627, 95% CI [-0.04, 0.07]	t(129) = -0.02, p = .986, 95% CI [-0.04, 0.04]	t(160) = 2.47, **p = .014**, 95% CI [0.01, 0.06]	t(157) = 0.17, p = .863, 95% CI [-0.03, 0.04]	t(172) = 1.23, p = .221, 95% CI [-0.01, 0.04]
	Statistical Test	Welch's t-test	t-test	t-test	t-test	t-test	t-test
	size effect	d = 0.4	d = 0.09	d = 0	d = 0.4	d = 0.03	d = 0.19
	**Severe Hearing Loss**						
		**Latencies (females)**			**Latencies (males)**		
		**I**	**III**	**V**	**I**	**III**	**V**
t-test	THI Score≥48 Vs Score<48	t(10,45) = 0.98, p = .348, 95% CI [-0.28, 0.72]	t(20) = 2.61, **p = .017**, 95% CI [0.06, 0.54]	t(20) = 2.43, **p = .025**, 95% CI [0.06, 0.79]	t(56) = -0.82, p = .415, 95% CI [-0.26, 0.11]	t(51) = -2.15, **p = .036**, 95% CI [-0.39, -0.01]	t(60) = 0.03, p = .975, 95% CI [-0.29, 0.3]
	Statistical Test	Welch's t-test	t-test	t-test	t-test	t-test	t-test
	size effect	d = 0.5	d = 1.12	d = 1.04	d = 0.22	d = 0.6	d = 0.01
		**Amplitudes (females)**			**Amplitudes (males)**		
		**I**	**III**	**V**	**I**	**III**	**V**
t-test	THI Score≥48 Vs	t(18) = -1.09, p = .29,	t(20) = -2.26, **p = .035**,	t(12.67) = -1.46, p = .169,	t(56) = -0.31, p = .761,	t(51) = 0.26, p = .792,	t(60) = 0.94, p = .352,

	Score<48	95% CI [-0.11, 0.04]	95% CI [-0.18, -0.01]	95% CI [-0.16, 0.03]	95% CI [-0.04, 0.03]	95% CI [-0.06, 0.07]	95% CI [-0.03, 0.07]
	Statistical Test	t-test	t-test	Welch's t-test	t-test	t-test	t-test
	size effect	d = 0.50	d = 0.97	d = 0.62	d = 0.08	d = 0.07	d = 0.24

Table S9. Statistical analyses regarding the level of tinnitus distress on the components of the AMLR waveforms in people with common hearing levels and gender.

		**Normal Hearing**							
		**Latencies (females)**				**Latencies (males)**			
		**Na**	**Pa**	**Nb**	**Pb**	**Na**	**Pa**	**Nb**	**Pb**
t-test	THI Score≥48 Vs Score<48	t(39) = -0.57, p = .574, 95% CI [-2.83, 1.59]	t(39) = -1.13, p = .264, 95% CI [-3.38, 0.95]	t(39) = -2,13, **p = .04**, 95% CI [-7.16, -0.18]	t(23.54) = -2.97, **p = .007**, 95% CI [-10.34, -1.86]	t(51) = 1.15, p = .255, 95% CI [-0.97, 3.57]	t(30,51) = 1.62, p = .115, 95% CI [-0.7, 6.09]	t(51) = 0.08, p = .937, 95% CI [-4.2, 4.54]	t(51) = 0.21, p = .836, 95% CI [-3.83, 4.71]
	Statistical Test	t-test	t-test	t-test	Welch's t-test	t-test	Welch's t-test	t-test	t-test
	size effect	d = 0.18	d = 0.36	d = 0.68	d = 1.04	d = 0.32	d = 0.45	d = 0.02	d = 0.06
		**Amplitudes (females)**				**Amplitudes (males)**			
		**Na**	**Pa**	**Nb**	**Pb**	**Na**	**Pa**	**Nb**	**Pb**
t-test	THI Score≥48 Vs Score<48	t(39) = -0.25, p = .8, 95% CI [-0.23, 0.18]	t(39) = -0.62, p = .538, 95% CI [-0.19, 0.1]	t(39) = -0.33, p = .746, 95% CI [-0.19, 0.13]	t(37.12) = -0.1, p = .924, 95% CI [-0.19, 0.18]	t(51) = -1.21, p = .232, 95% CI [-0.36, 0.09]	t(51) = 0.77, p = .446, 95% CI [-0.11, 0.26]	t(51) = 0.94, p = .352, 95% CI [-0.08, 0.23]	t(51) = 1.98, p = .053, 95% CI [-0.01, 0.31]
	Statistical Test	t-test	t-test	t-test	Welch's t-test	t-test	t-test	t-test	t-test
	size effect	d = 0.08	d = 0.2	d = 0.1	d = 0.03	d = 0.34	d = 0.21	d = 0.26	d = 0.55
		**Mild Hearing Loss**							
		**Latencies (females)**				**Latencies (males)**			
		**Na**	**Pa**	**Nb**	**Pb**	**Na**	**Pa**	**Nb**	**Pb**
t-test	THI Score≥48 Vs Score<48	t(110) = 1.72, p = .088, 95% CI [-0.2, 2.77]	t(110) = 0.77, p = .442, 95% CI [-1.03, 2.35]	t(110) = 0.66, p = .51, 95% CI [-1.69, 3.39]	t(110) = 1.31, p = .194, 95% CI [-1.05, 5.1]	t(161) = 0.57, p = .568, 95% CI [-1.02, 1.85]	t(164) = 1.98, p = .05, 95% CI [-0.01, 2,85]	t(164) = 2, **p = .047**, 95% CI [0.02, 3.96]	t(112.58) = 1.21, p = .231, 95% CI [-0.92, 3.76]
	Statistical Test	t-test	t-test	t-test	t-test	t-test	t-test	t-test	Welch's t-test
	size effect	d = 0.33	d = 0.15	d = 0.13	d = 0.25	d = 0.09	d = 0.32	d = 0.32	d = 0.19
		**Amplitudes (females)**				**Amplitudes (males)**			
		**Na**	**Pa**	**Nb**	**Pb**	**Na**	**Pa**	**Nb**	**Pb**
t-test	THI Score≥48 Vs Score<48	t(110) = -0.9, p = .37, 95% CI [-0.2, 0.07]	t(110) = 2.03, **p = .044**, 95% CI [0, 0.24]	t(110) = -3.13, **p = .002**, 95% CI [-0.27, -0.06]	t(110) = 1.96, p = .052, 95% CI [-0.01, 0.24]	t(115.37) = -3.64, **p <.001**, 95% CI [-0.25, -0.07]	t(164) = 3.85, **p <.001**, 95% CI [0.07, 0.22]	t(164) = -2.37, **p = .019**, 95% CI [-0.17, -0.01]	t(164) = 0.84, p = .403, 95% CI [-0.05, 0.12]
	Statistical Test	t-test	t-test	t-test	t-test	Welch's t-test	t-test	t-test	t-test

	size effect	d = 0.17	d = 0.39	d = 0.61	d = 0.37	d = 0.59	d = 0.61	d = 0.38	d = 0.13
	**Severe Hearing Loss**								
		**Latencies (females)**				**Latencies (males)**			
		**Na**	**Pa**	**Nb**	**Pb**	**Na**	**Pa**	**Nb**	**Pb**
t-test	THI Score≥48 Vs Score<48	t(18) = 0.47, p = .641, 95% CI [-3.02, 4.79]	t(19) = -1,31, p =.207, 95% CI [-5.2, 1.2]	t(19) = -0.14, p = .89, 95% CI [-5.87, 5.14]	t(19) = 0.16, p = .873, 95% CI [-5.86, 6.85]	t(59) = 1.16, p = .252, 95% CI [-0.98, 3.66]	t(59) = 1.42, p = .161, 95% CI [-0.72, 4.27]	t(59) = 0.46, p = .646, 95% CI [-2.53, 4.04]	t(59) = 0.7, p = .486, 95% CI [-2.52, 5.25]
	Statistical Test	t-test	t-test	t-test	t-test	t-test	t-test	t-test	t-test
	size effect	d = 0.22	d = 0.58	d = 0.06	d = 0.07	d = 0.3	d = 0.37	d = 0.12	d = 0.18
		**Amplitudes (females)**				**Amplitudes (males)**			
		**Na**	**Pa**	**Nb**	**Pb**	**Na**	**Pa**	**Nb**	**Pb**
t-test	THI Score≥48 Vs Score<48	t(18) = 1.77, p = .094, 95% CI [-0.04, 0.52]	t(16.26) = -0.97, p = .348, 95% CI [-0.32, 0.12]	t(14.18) = 0.89, p =.39, 95% CI [-0.15, 0.36]	t(19) = -0.3, p = .768, 95% CI [-0.31, 0.23]	t(59) = -2.51, **p = .015**, 95% CI [-0.28, -0.03]	t(56.9) = 1.56, p = .124, 95% CI [-0.02, 0.15]	t(59) = -0.76, p = .453, 95% CI [-0.17, 0.08]	t(59) = 1.67, p = .101, 95% CI [-0.02, 0.21]
	Statistical Test	t-test	t-test	t-test	t-test	t-test	Welch's t-test	t-test	t-test
	size effect	d = 0.81	d = 0.39	d = 0.35	d = 0.13	d = 0.65	d = 0.4	d = 0.19	d = 0.43

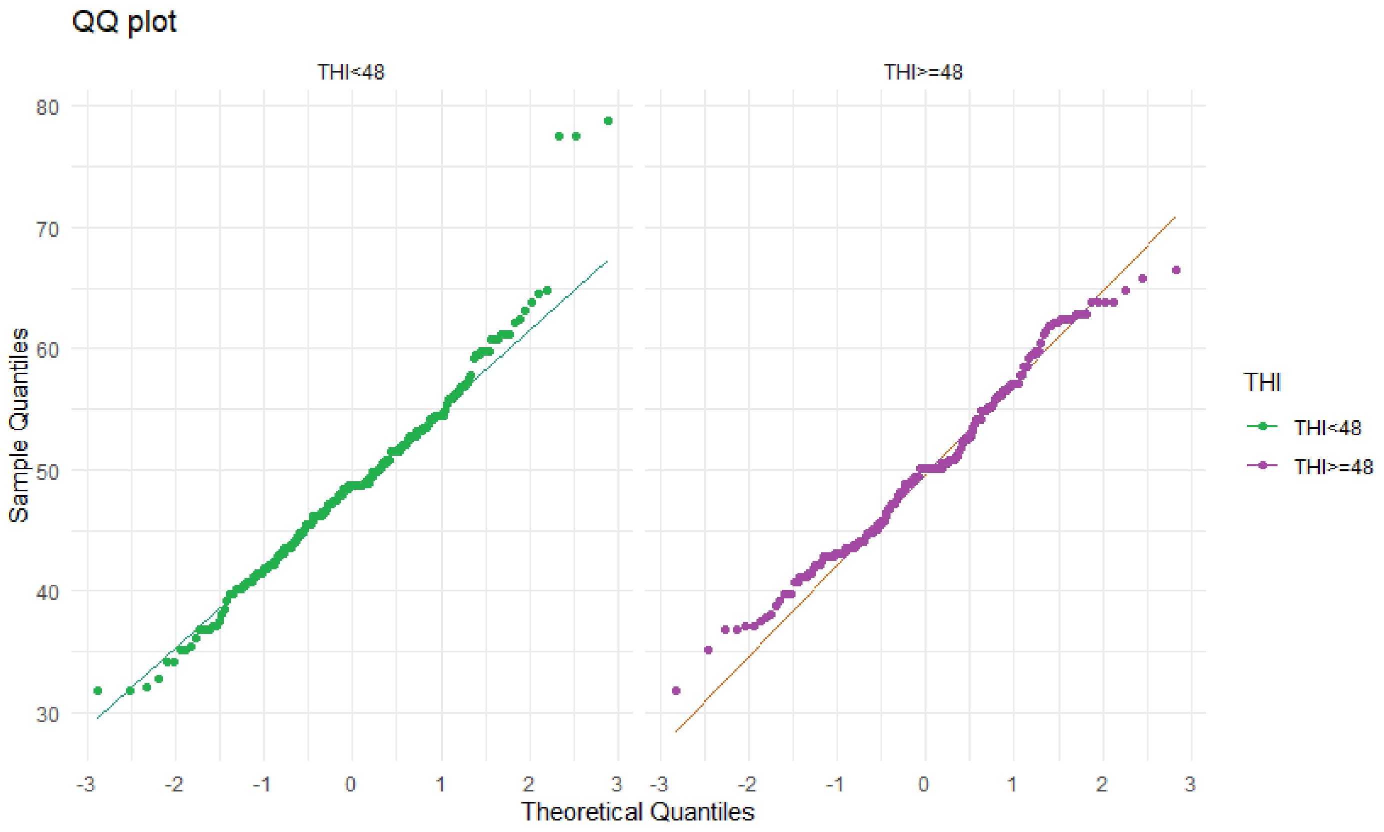

Figure S1. The QQ plot was used to test whether the dependent variable "Pb latency" followed a normal distribution.

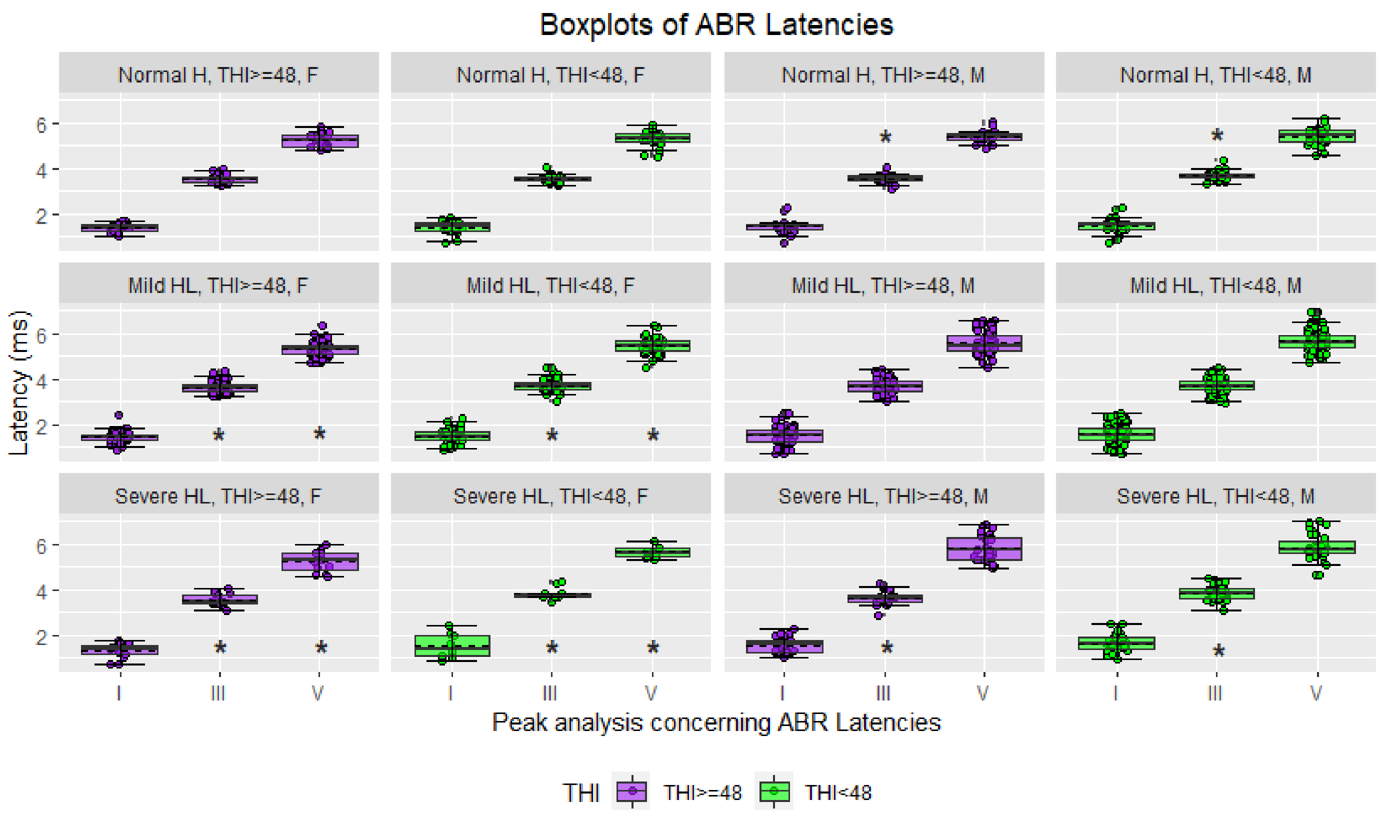

Figure S2. Boxplots of ABR waveforms latencies based on tinnitus distress in people with common hearing levels and gender (in purple: severe/high tinnitus distress; in green: mild/moderate tinnitus distress; H=hearing; HL=hearing loss; M=males and F=females; asterisks indicate significance: * *p*-value ≤ 0.05; ** *p*-value ≤ 0.01; *** *p*-value ≤ 0.001).

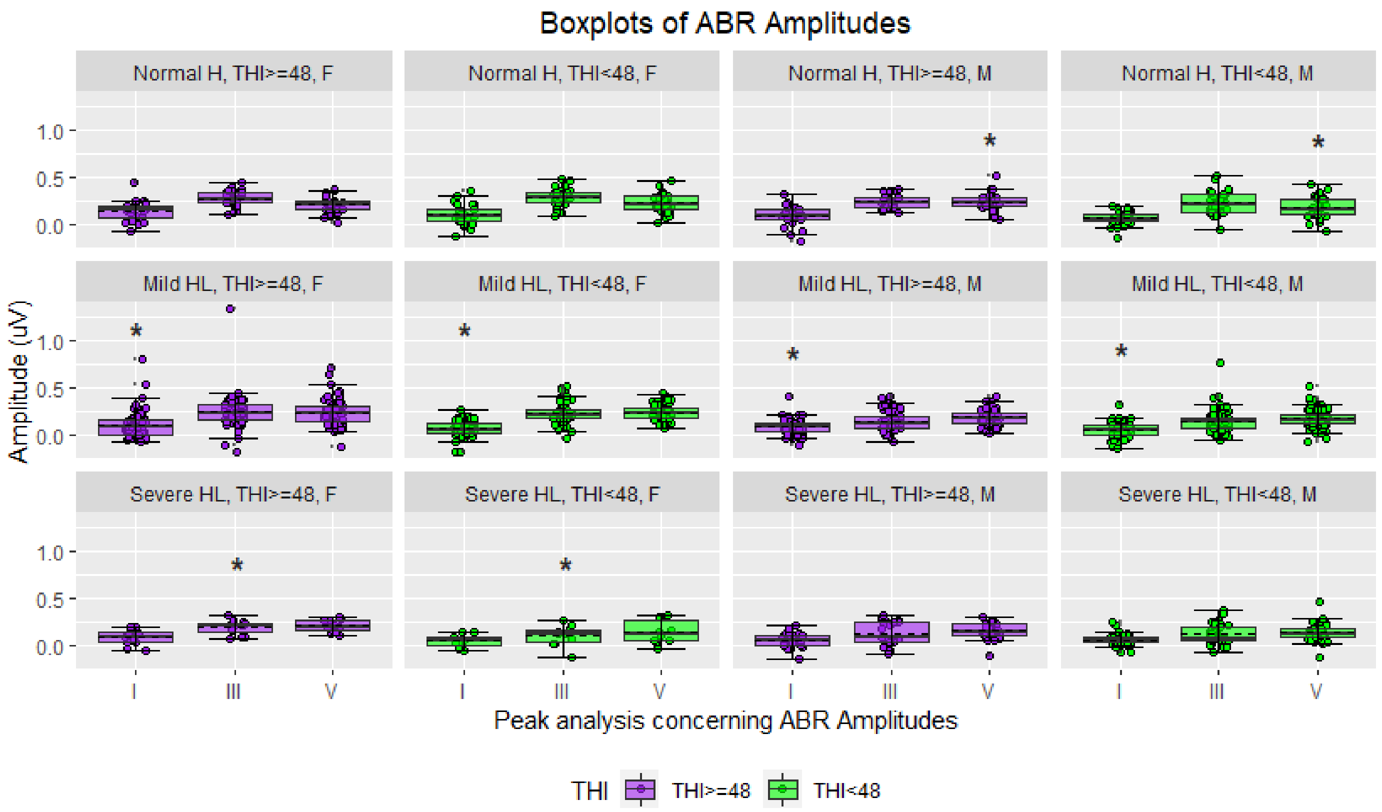

Figure S3. Boxplots of ABR waveforms amplitudes based on tinnitus distress in people with common hearing levels and gender (in purple: severe tinnitus distress; in green: mild tinnitus distress; H=hearing; HL=hearing loss; M=males and F=females; asterisks indicate significance: * *p*-value ≤ 0.05; ** *p*-value ≤ 0.01; *** *p*-value ≤ 0.001).

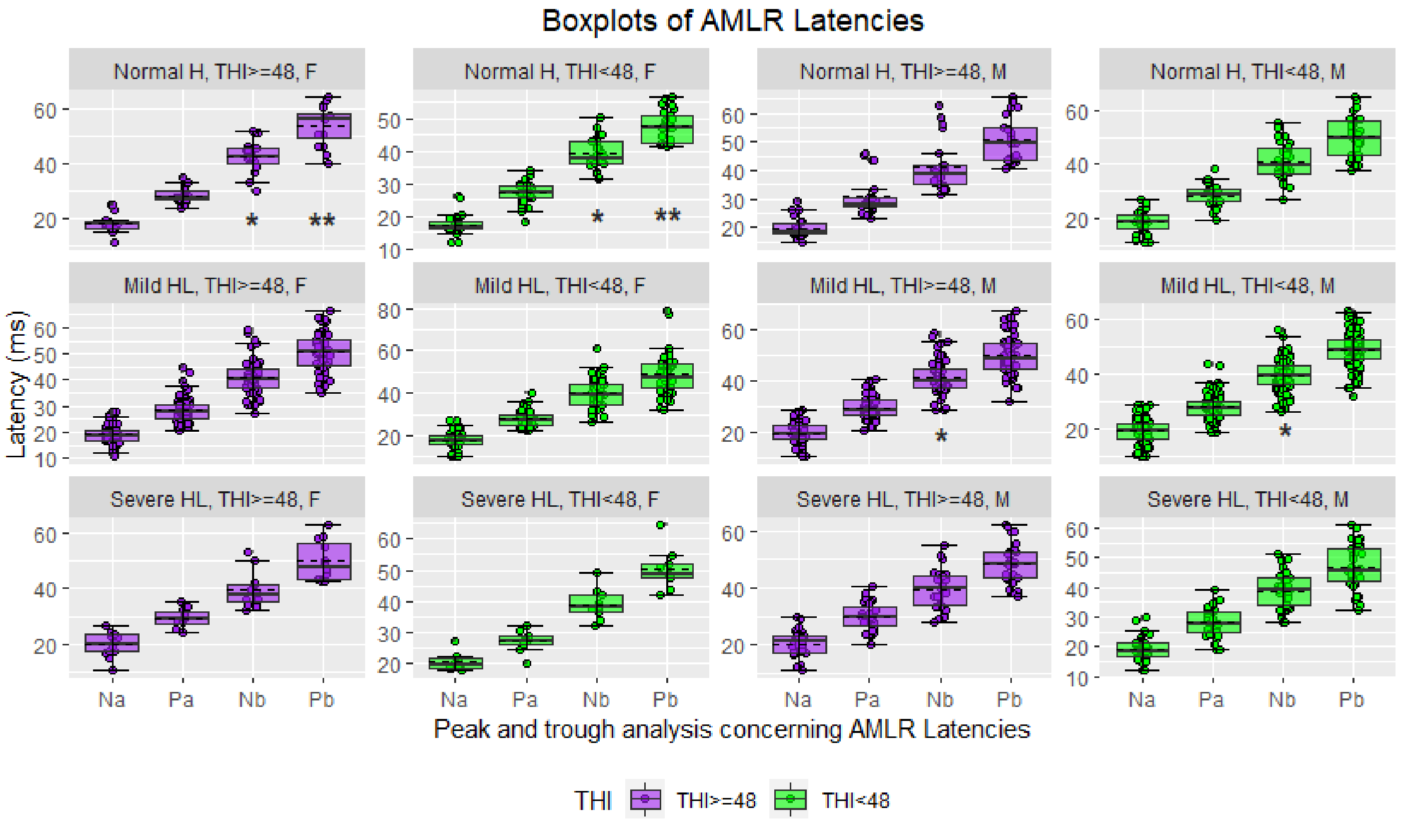

Figure S4. Boxplots of AMLR waveforms latencies based on tinnitus distress in people with common hearing levels and gender (in purple: severe/high tinnitus distress; in green: mild/moderate tinnitus distress; H=hearing; HL=hearing loss; M=males and F=females; asterisks indicate significance: * *p*-value ≤ 0.05; ** *p*-value ≤ 0.01; *** *p*-value ≤ 0.001).

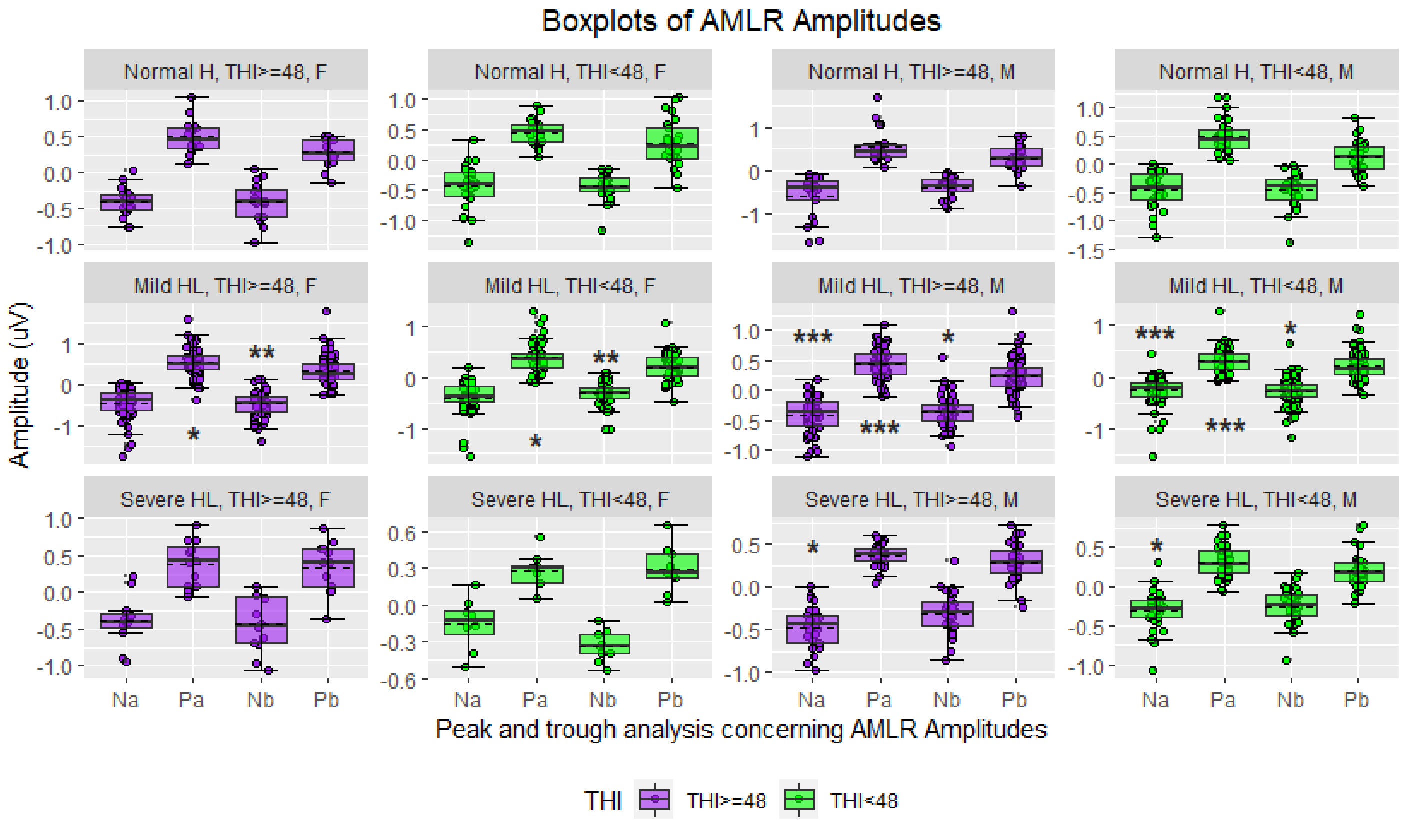

Figure S5. Boxplots of AMLR waveforms amplitudes based on tinnitus distress in people with common hearing levels and gender (in purple: severe/high tinnitus distress; in green: mild/moderate tinnitus distress; H=hearing; HL=hearing loss; M=males and F=females; asterisks indicate significance: * p-value ≤ 0.05; ** p-value ≤ 0.01; *** p-value ≤ 0.001).
